# SBTM: epileptic seizure prediction from EEG signal using deep learning in blockchain-enabled smart healthcare monitoring with IoT networking

**DOI:** 10.1038/s41598-026-36425-1

**Published:** 2026-01-31

**Authors:** Abhishek Kumar, Esha Tripathi, Abhay Kumar Tripathi, Himanshu Kumar Diwedi, Pramod Singh Rathore, Arshiya S. Ansari

**Affiliations:** 1 Department of Computer Science and Engineering, Chandigarh University, Gharuan,, Mohali, Punjab, 140413 India; 2 Department of Information Technology, Pranveer Singh Institute of Technology, Kanpur, India; 3 Department of Computer Science and Engineering, Pranveer Singh Institute of Technology, Kanpur, India; 4 Department of Information Technology, Pranveer Singh Institute of Technology, Kanpur, India; 5 Department of Computer and Communication Engineering, Manipal University Jaipur, Jaipur, India; 6 Department of Information Technology College of Computer and Information Sciences, Majmaah University, 11952 Al-Majmaah, Saudi Arabia

**Keywords:** IoT networking, Smart healthcare monitoring, Blockchain, Epilepsy seizure prediction, Deep learning, Biological techniques, Biotechnology

## Abstract

Epileptic Seizure prediction is highly significant for the identification and reduction of high risks related to serious brain injuries, strokes, and brain tumors. Early and accurate diagnosis is vital for providing intervention measures for enhancing the quality of life of the affected individuals. Numerous techniques have been developed based on Machine vision techniques to predict epileptic seizures. Nonetheless, the acquisition of precise epileptic seizure detection with low false positive rates is challenging. Moreover, the emergence of the Internet of Things (IoT) revolutionized healthcare monitoring with technological improvements, aiming to handle the concerns related to data interoperability, scalability, as well as privacy issues. Hence, this research proposes the Smart Healthcare Monitoring Framework, namely Spizella Optimization-based Bidirectional Short Term Memory Network (SBTM), for determining the seizure states, thereby allowing the provision of remote care. Specifically, the proposed model exploits the Bi-LSTM architecture that captures the temporal dependencies and nonlinear dynamics of EEG signals, making the model highly efficient for predicting the seizure patterns. Besides, the Spizella Optimization is applied for fine-tuning the hyperparameters of the classifier, thereby leading to accurate prediction. Experimental results demonstrate that the proposed SBTM model accomplishes superior results by achieving high accuracy, sensitivity, and specificity equivalent to 97.52%, 97.51% and 98.51% with 90% training, outperforming the state-of-the-art techniques. Moreover, the presented approach significantly improves the remote monitoring, guaranteeing on-time medical care, ensuring data security, and enhancing the overall performance of applications in tech-aided healthcare systems.

## Introduction

Information technology gathers essential information at a click, which plays a major role in applications like healthcare, industrial automation, agriculture, smart cities, smart homes, and several other factors. Among all the mentioned applications, healthcare stands as a significant unit in automation, making tremendous changes in the smart healthcare sector improvement^1^. With the use of tracking and sensing devices implanted in the human body, smart healthcare systems can monitor and diagnose patients’ vital signs, including heart rate, blood sugar, electroencephalogram (EEG), electrocardiogram (ECG), and other vital biological data^1,2^. Medical conflicts can be resolved with the help of EHR, which offers the most comprehensive and practical data for diagnosing and identifying a range of diseases^3^. The Internet of Things (IoT) has recently captured a lot of interest due to the previously described qualities in healthcare^1^. The goal of a smart healthcare system is undoubtedly the way of the future, given its enormous market and user base. However, there are a number of internal and external social and technological obstructions that must be overcome for this SHS to reach the population’s center in the most remote places. Common societal limiting issues in rural locations include a lack of information, resistance to adopting new technologies, conventional and irrational thinking, and the absence of basic infrastructure. Smart healthcare faces challenges, such as privacy and security threats, which must be solved for health quality^2^. Thus, the Blockchain is utilized for the trust healthcare environment with characteristic features, such as a decentralized nature and gradient mining based on the tamper-proof and trustworthy aggregator. The data sharing and retrieving based on the blockchain makes transparent, privacy-preserved, and secure communication concerning the smart healthcare environment^4^.

The increasing population attracts smart healthcare, and the advancements, for which IoT plays a prominent role, particularly in the epileptic seizure prediction for the enhancement of the patient’s life quality^5,6^. The necessity of the blockchain in smart healthcare is the management of electronic health records (EHR). Epilepsy seizure is a life-threatening disease that may happen anytime and anywhere while walking, driving, running, sleeping, and so on, thus, the smart healthcare management of these patients is still a challenging task^7,8^. Nevertheless, at the time of a seizure, the EEG signals display distinctive patterns that imitate abnormal neuronal activity with high frequency and amplitude^9,10^. Thus, constant monitoring is necessary for updating the sufferer’s health status, which requires an authenticated device. So, the blockchain is utilized for storing the patient’s information and can be used for analysis as per the requirement^11^. The lack of skilled manpower, security, and privacy concerns of data sharing are considered a challenging task. The applications of blockchain are progressing every day in the healthcare sector for sharing patient information. The blockchain is utilized in several ways as per the requirement to satisfy the need^12^. The security and privacy of the blockchain network make it utilized in several fields with the availability, flexibility, and scalability factors. Due to the availability of these factors, the blockchain is incorporated with the Machine Learning and Deep Learning techniques for overcoming the challenges^13,14^. The information stored in the blockchain ensures authenticity and can be utilized by machine learning techniques for future verification with enhanced accuracy^15^^10,16^.

To deal with the concerns in the existing techniques, the presented research presents the IoT-assisted Epileptic seizure prediction using the SBTM model that attempts to apply blockchain and IoT technologies in smart healthcare systems to suggest on-time diagnosis for patients with better accuracy when compared with manual diagnosis, which consumes time and suffers from inaccuracy issues. Moreover, the monitoring of the registered physicians is enabled department-wise, and recognized under the genuine user category for accessing the patient data of their own department for diagnosing the disease. Thus, the focus on authenticated data access and patient health monitoring supports the disease detection accuracy.

Due to the authenticated data communication and storage in smart healthcare systems, Blockchain technology is used in this work along with the deep learning model. The diagnosis support is assisted by the DL model for rendering accurate predictions for which the SBTM model is designed, which improves the prediction accuracy with better maintenance of intensification and diversification, thereby minimizing the training loss. The research’s major contributions are:

***Smart Healthcare Monitoring using Blockchain Technology:*** The patients are monitored from a remote location to provide on-time medical assistance to promote the survival rate. In this instance, the sensors engaged in collecting the patient information are communicated over the hospital portal for the doctor’s perusal, and for ensuring the secure transmission and storage of the sensitive medical data, Blockchain technology is employed that ensures double-layer protection against unauthorized data access.

***Spizella Optimization:*** The Spizella optimization hybridizes the unique foraging characteristics of the Spizella by considering the alarm indication and the striving behavior of the cougar to reach the optimal solution. Further, the Spizella optimizer effectively explores the large search space using the global searching ability and offers the global optimal solution for tuning the SBTM model, improving the epileptic seizure prediction.

***Proposed IoT-assisted Blockchain-enabled Spizella Optimized BiLSTM Network (SBTM):*** In the proposed research, the BiLSTM model effectively gathers the temporal dependencies and nonlinear dynamics of EEG signals in sequential data by exploiting both the forward and backward flow of information. Further, the Spizella Optimization adaptively fine-tunes the hyperparameters of the SBTM classifier, resulting in enhanced prediction accuracy.

The remainder of the research is arranged as follows: The related studies, along with research gaps, are presented in Motivation, and System model of IoT-blockchain based epileptic seizure prediction elucidates the proposed methodology. The evaluations are represented in Result and discussion, whereas Conclusion concludes the research work with future directions.

## Motivation

The review of the conventional Epilepsy seizure prediction techniques based on machine learning and smart healthcare faces several challenges that limit the performance enhancement, which motivates to development of a novel smart healthcare-based epilepsy seizure prediction technique.

### Related works

Dhanalekshmi Prasad Yedurkar et al.^17^ implemented the IoT-enabled EEG monitoring system utilizing the Convolutional Neural Network (CNN) method for identifying EEG seizures. In addition, the epileptic information obtained from the multichannel EEG data associated with many brain regions offers valuable information for improving performance. Further, the system leveraged critical spectral verge (CSV) features obtained using the spike-statistical (SS) flower pollination algorithm (FPA) for improving the epileptic seizure prediction. Banu Priya Prathaban et al.^18^ introduced the IoMT-based headband ForeSeiz for epileptic seizure prediction. Specifically, the ForeSeiz involves the Enhanced Convolutional Neural Network (ECNN) model, optimally tuned utilizing the Fletcher Reeves Algorithm for enhancing the convergence and reducing the model complexity. However, the IoT-constrained device hardware and a simple architecture with less complexity were required for this framework to work with real-time scenarios.

Kuldeep Singh and Jyoteesh Malhotra^19^, implemented the automatic epileptic seizure detection system utilizing communication technologies in association with ML and cloud computing for improving the detection of epileptic seizures. Specifically, the Random Forest (RF) technique was applied for the EEG signal classification. For highlighting the performance of the RF model, other ML algorithms, including the Bayes Net and Multilayer Perceptron, were utilized for analyzing the performance. However, the RF often struggles in analyzing the high-dimensional data and was observed with slower inference times for processing the large number of trees. Ankur Goyal et al.^20^ introduced the Internet of Things (IoT) framework utilizing the Artificial Neural Network (ANN) and PSO algorithm for boosting the physiological sensor-data fusion. Besides, the PSO-ANN approach assisted in the automatic identification of epilepsy and brain fatalities from the EEG signals received by the health center. In addition, the particle swarm computation assists in optimizing the propagation of neural networks and EEG. Further, the healthcare services were extremely delay-sensitive services, and required an energy-efficient IoT-based cloud network.

M. Ramkumar et al.^21^ presented the IoT-enabled automated epileptic seizure detection framework utilizing the Siamese Convolutional Fire Hawk Sparse Autoencoder Network (SCFHSAN) model. In the SCFHSAN model, the preprocessing for artifact removal was carried out using adaptive filtering and multi-resolutional analysis. In addition, the dandelion tunable Q-wavelet transform was applied for decomposing the signals into frequency sub-bands. Ultimately, the fire hawk optimization algorithm was utilized for optimally tuning the hyperparameters of the network. Kunpeng Song et al.^22^ developed the Intelligent Epileptic Prediction System utilizing the Synchrosqueezed Wavelet Transform (SWT) and Multi-Level Feature Convolutional Neural Network (MLF-CNN) for a smart healthcare IoT network. Additionally, the MLF-CNN model was recognized for automatically extracting the features from different dimensions. Nevertheless, the model showcased poor performance for a few patients. Although the MLF-CNN model offers better performance, with a small sample size, which possibly limits the generalizability for the diverse dataset.

Ali Kadhum Idrees et al.^23^ introduced the Internet of Medical Things (IoMT) based epileptic seizure prediction, which forwarded the huge volume of sensed data to the data centers of the cloud for treatment. In addition, the method of epileptic seizure detector-based Naive Bayes (ESDNB) algorithm was applied, which improved prediction. Specifically, the model minimized the size of IoMT EEG data distributed to the cloud via the application of the same lossless compression algorithm. Furthermore, the lossless compression technique would be enhanced to improve the compression power. Hisham Daoud et al*.*^24^ suggested the IoT framework using a deep learning network for precise epileptic seizure prediction. In the deep learning model, both the feature extraction and classification stages were integrated for processing the raw EEG signals without any preprocessing, minimizing the computation complexity. More specifically, the CNN-based model captured the significant spatio-temporal features from the EEG signals. Besides, the channel selection algorithm was utilized, which minimized the complexity and the memory required for the system to accomplish the real-time application.

Jagadeesh Basavaiah et al.^25^ introduced the Machine Learning (ML) based remote monitoring system utilizing several sensors and equipment, including the ECG sensor, tilt, GPS, and GSM module, and vibration sensors for improving the prediction of epilepsy. In addition, the system provides alerts when the patient is undergoing a seizure, allowing timely intervention and treatment. However, the model requires enhancing the ML model by incorporating additional features and fine-tuning the hyperparameters. Nevertheless, the model requires combining blockchain technology to increase the security and confidentiality of the patient’s information.

Mohamadreza Khosrav*, *et al*.*^26^*,* presented an attention-based model for processing the EEG signals in a medical support system. To facilitate targeted analysis, the EEG signals were transformed to distinct frequency bands, and the dynamic nature was handled using smaller windows. The distribution over frequency and time was visualized; however, the computational requirements were higher, which hindered the applicability in real-time clinical practice. Rezaee Kh*, *et al*.*^27^*,* suggested an optimized algorithm for seizure detection implemented using discriminant analysis. The wavelet transform was employed to visualize the temporal and spectral spaces, providing more adaptability in determining the mapping matrix. Furthermore, the feature vectors capable of discriminating different classes were acquired from the high-dimensional space, nonetheless, the error rates were higher for samples with subtle abnormalities.

Shabnam Hesari*, *et al*.*^28^*,* presented an ensemble feature learning method that supported multi-faceted processing in EEG signal analysis. The waveform was transformed to relevant subbands, and the non-stationarity of the signals was handled through signal windowing. The subbands of the signal were interpreted individually to obtain intrinsic and high-dimensional representations. However, the lack of data diversity affected the model’s generalization to unseen samples.

### Challenges

The challenges faced by conventional seizure prediction methods are.

- Although the MLF-CNN model provides better performance, the small size of the sample affected the generalizability of the diverse dataset^22^.
- In addition, the ML-based remote monitoring system requires improving the ML model by integrating additional features and fine-tuning the hyperparameters. Nevertheless, the ML model requires combining blockchain technology for the enhancement of security and confidentiality related to patient information^25^.
- The classification complexity increased with the increasing dimension of the features. In addition, the existing techniques do not consider the unique spatial–temporal features related to EEG, resulting in limited performance^15^.
- Epileptic seizures were found to exist in various complex patterns, making it challenging to accurately detect all types of seizures^29^.
- Another issue related to capturing irrelevant details and noise happens when the model is overfitting using the training data. This results in a high number of trainable parameters^30^.

Most of the existing seizure prediction models suffer from the need for high computational time and substantial memory resources. Also, there is a risk of overfitting due to a high number of trainable parameters. In addition, the existing model requires combining blockchain technology to boost the security and confidentiality level of patient information. Specifically, the existing methods faced complexity with the increasing dimension of the features. In addition, the existing techniques fail to consider the unique spatial–temporal features of EEG, resulting in limited performance. Epileptic seizures were found to exist in different intricate patterns, making it challenging to accurately detect all types of seizures. In contrast, the proposed model leverages Blockchain with Spizella BiLSTM predictor to address these issues. More precisely, the proposed model exploits the BiLSTM architecture in capturing temporal dependencies and nonlinear dynamics of EEG signals, making the model highly efficient for learning the variability in the complex patterns associated with epileptic seizures. Besides, the Spizella Optimization is applied for fine-tuning the hyperparameters of the classifier, resulting in improved prediction accuracy.

## System model of IoT-blockchain based epileptic seizure prediction

**Fig. 1:**
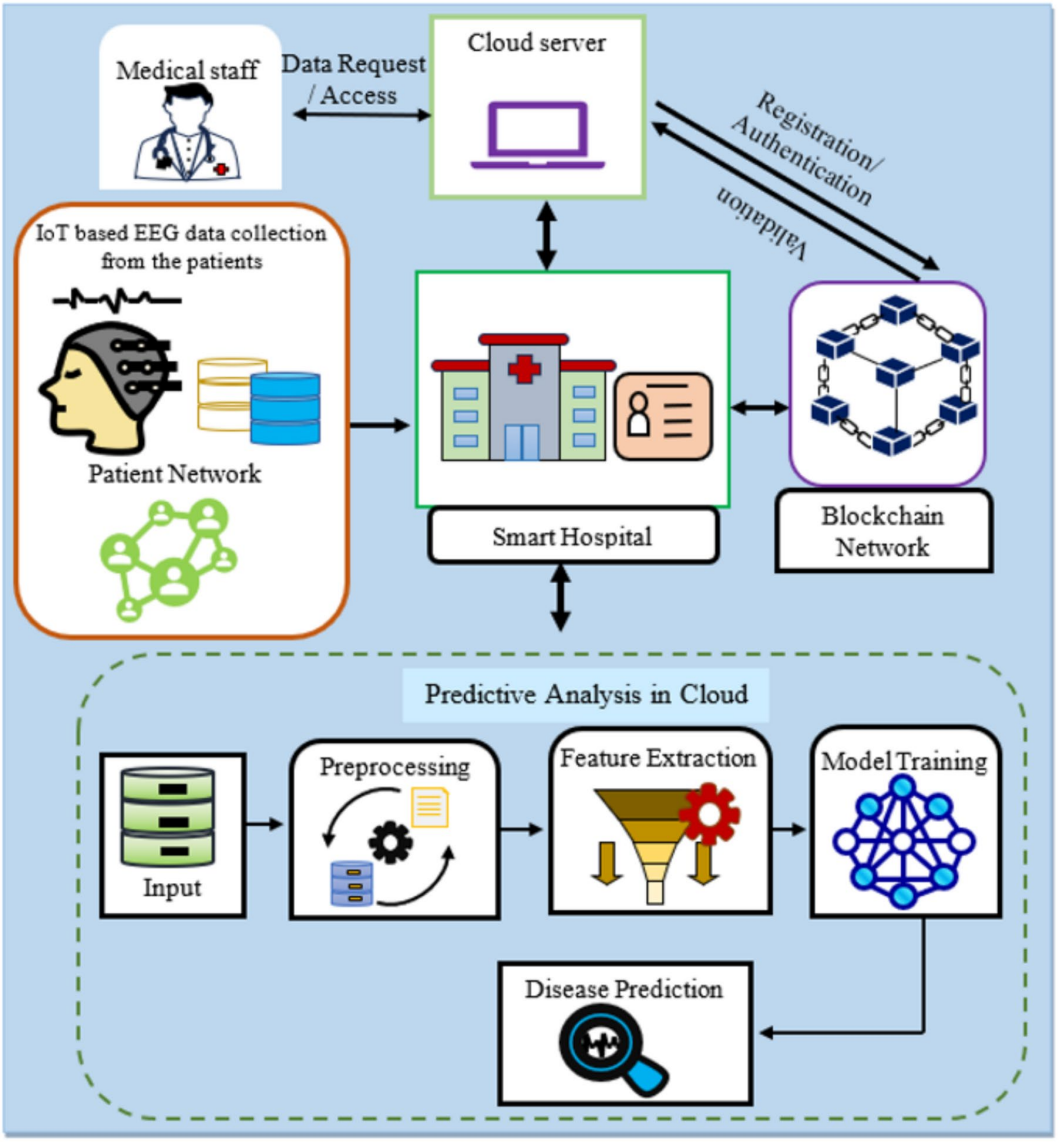
System model for the IoT-blockchain-based epileptic seizure prediction.

In recent decades, IoT-assisted smart healthcare systems technology has grown rapidly in the field of healthcare. As this sector broadens, it necessitates doorstep diagnosis, easy.

surveillance and controlling the data to handle complexities in data interoperability, scalability, and security. Specifically, the main intention is to immerse the IoT in urgent services by connecting the IoT devices and sensors to exchange healthcare information. Consequently, these data are collected with the help of intelligent devices and an intelligent hospital for monitoring the disease indicators in real-time. However, the IoT-based health system integrates tools, sensor monitors, and transfers health-related information over insecure public channels, resulting in many security constraints like impersonation, eavesdropping, and malicious attacks. Despite the progress offered by the IoT-aided systems, Epileptic seizure detection inherits several potential challenges, ranging from the complexity of EEG signals to the need for real-time monitoring. One of the key challenges includes variability in seizure patterns. Epileptic seizures often present in distinct and complex patterns, making it challenging to accurately detect all types of seizures. Hence, the research presents the IoT-based blockchain-enabled Optimal deep learning with an authentication mechanism for secure Epileptic Seizure prediction. The system model for the IoT-blockchain-based epileptic seizure prediction is depicted in Fig. 1. The system portrays the communication between several communicating devices, including the medical IoT gadgets, blockchain storage, and optimized DL models for epileptic seizure prediction. Hence, the patient records are stored in a tamper-resistant and transparent manner on the blockchain in the proposed IoT-based epileptic seizure prediction. When a medical practitioner desires to acquire a patient’s information, they initiate a formal request through the hospital’s system, which is processed by the blockchain network, ensuring authorized individuals have access to the data. This setup enhances data security, transparency, and access control in the healthcare environment, ultimately benefiting both patients and medical professionals. The communications are handled through Blockchain technology to enable secure data access. Moreover, the prediction is supported by the automatic decision support system developed using deep learning. In the proposed system, the simulated patient network is executed through Blockchain technology. The data is stored in the hospital portals, which are organized as different health departments headed by physicians. This system ensures on-time diagnosis through the application of IoT and blockchain technologies, for which the patient’s information is communicated with specialized health professionals, who are recognized as genuine users in the blockchain system. Finally, the health data in the hospital portals is suggested for the epileptic seizure diagnosis, for which a deep learning framework is proposed, resulting in improved prediction accuracy.

### Smart healthcare system – data acquisition

The patient’s EEG is collected and controlled using IoT and blockchain technologies, which is processed by the genuine physicians to suggest the diagnosis. EEG records electrical signals from the scalp, directly measuring the brainwaves that provide real-time insights into brain function and detect the abnormal patterns associated with seizures. Seizure prediction using EEG can considerably improve the quality of individuals with epilepsy. Providing warnings helps the patients and doctors take precautionary measures, potentially preventing injuries and improving overall well-being.

The blockchain-driven disease diagnosis approach employs IoT sensors for EEG signal acquisition from patients. These acquired EEG signals are subsequently stored within the blockchain by the designated service provider. Consider a scenario where $n$ denotes the number of patients within the hospital network. In this blockchain ecosystem, each hospital functions as an individual block. Consequently, $m$ denotes the total count of interconnected hospitals and $d$ shows the department within the blockchain network. In this proposed methodology, the Ethereum blockchain is selected for its decentralized attributes and secure data exchange capabilities facilitated by smart contracts. The EEG from $n$ patients is communicated to the hospital server through the blockchain is represented as

1 $$r = \{ r_{1} ,r_{2} ,....r_{l} ,.....r_{m} \}$$

where the total number of samples is referred as $m$, and $l$ indicates the randomly chosen sample from the total samples.

### Blockchain and data authentication for enabling authorized access

**Fig. 2:**
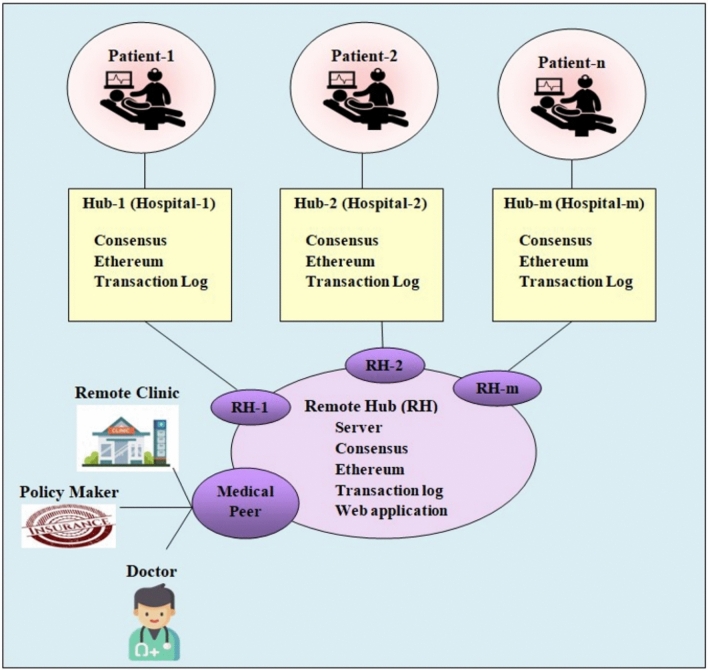
Architecture of Blockchain-based data storage- A single Hospital Network.

The data is stored in the respective hospital portals that are protected using the protective encryption keys, which are required to decrypt the data during server load. The blockchain secures the data storage through a decentralized architecture with cryptographic techniques, where the patients are allowed to store their health reports in the storage system, for which the patient is provided with a unique ID and password. Therefore, each time the patient data is stored in the same portal ID, the portal belongs to different hospitals with organized departments headed by specialists. Whenever the doctor needs access to the patient data, the doctor signs into the portal using their identity and proves their genuine doctor index. Once the doctor is recognized, data access is provided. It is peculiar to note that the data of the patient is saved in their respective departments, headed by the respective doctors. Once their identity is approved by the access control schemes, then data access is granted, for which address keys are provided to unlock the encrypted patient data. The storage process in blockchain is systematically presented in Fig. 2.

#### Patient network:

The patient network includes the patients, who are registered within a hospital’s portal system, and hold their unique identification on the network. The patient networks consist of health records of the patients with their personal data. Networks offer an efficient way to share patient information with various accomplices in the healthcare system. In this context, the records of EEG signals of different patients are used to predict seizures.

#### Blockchain network

The blockchain network is a decentralized and secure ledger technology that stores and manages information across distributed hospital portals to securely transmit the data from one end to another.

#### Storage unit (hospital portals)

The storage unit in this context refers to the digital infrastructure, where the hospital stores the patient information, which is typically accessible through hospital portals, where medical records and other relevant information belonging to the patients are organized under their unique identity.

#### Data users

Doctors within the healthcare system are responsible for diagnosing and treating patients, and genuine doctors hold a unique identity, including their specialization and login ID. The keys play a prominent role in releasing the patient data, and such keys are dynamically managed through the blockchain. The doctors entering their unique identity and passing the authentication through the Blockchain are regarded as genuine users, who encrypt the data using the keys and access the data to suggest any disease diagnosis.

#### Data request

Doctors, when they need to access a patient’s medical records or other relevant information, initiate a data request. This is a formal request made through the hospital’s system to retrieve specific patient data. The request consists of the information, such as the doctor’s registered ID $D_{ID}$, department $d$ , and specialization $s$. Using the accessed data, the diagnosis is set, and, in this research, the EEG signal of the patient is accessed for predicting the seizures in the patient and avoiding huge losses through the smart health support.

### Disease prediction framework using SBTM model for epilepsy seizure prediction

The proposed blockchain-based disease diagnosis system based on the Bi-LSTM provides a more accurate prediction for the continuous remote techniques due to the insensitivity concerning the gap length. The EEG signal acquired from the patient using the sensor is stored in the local hub, which means the hospital server. Then, the data is further saved in the blockchain so that it can be accessed as per the requirement anytime and from anywhere. Using the accessed health records, the doctor moves with the disease diagnosis for which the raw EEG signal is processed to extract the information that is analyzed using the Spizella-BiLSTM predictor for predicting the seizures.

**Fig. 3:**
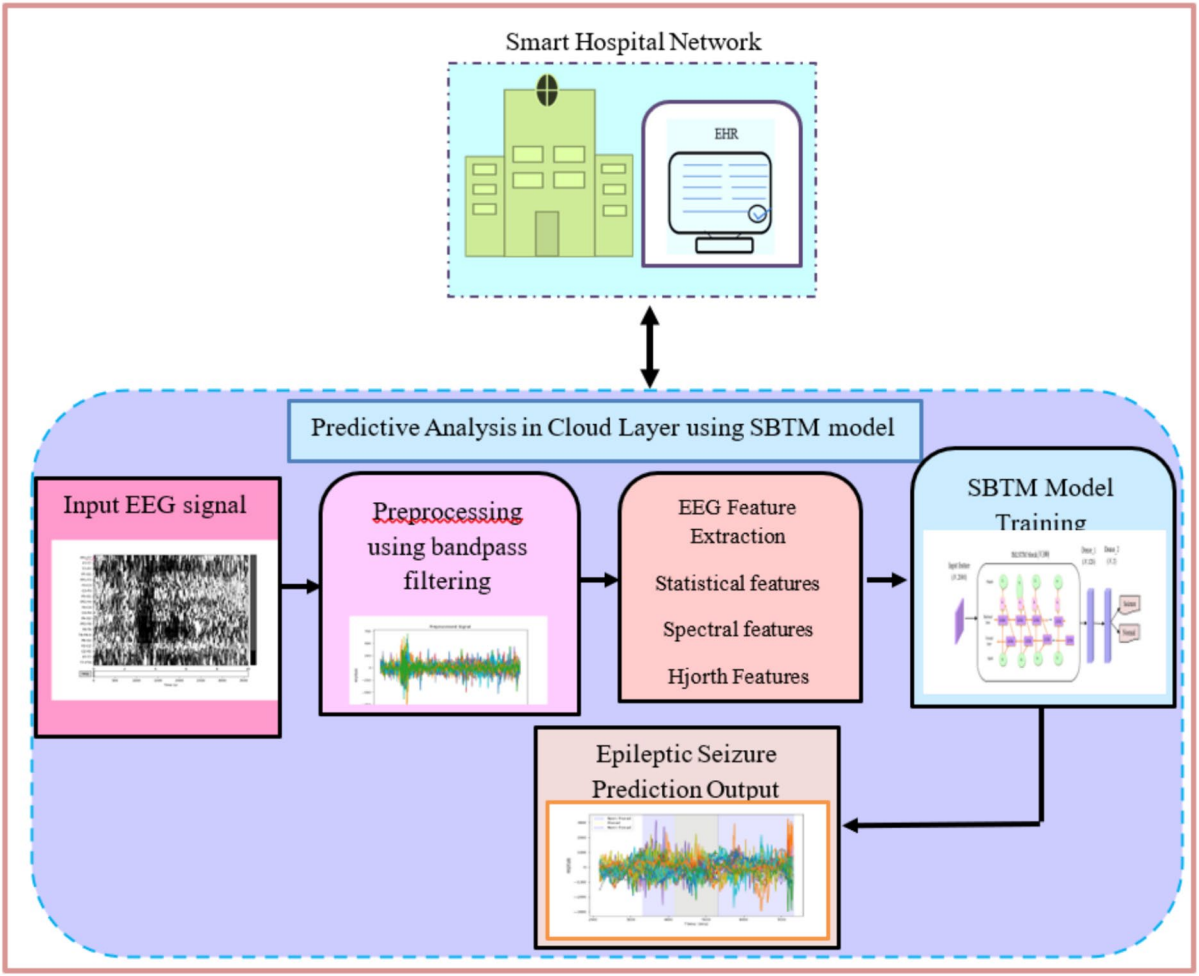
Proposed IoT-assisted Blockchain-enabled SBTM model for Epileptic seizure Prediction.

From the retrieved EEG signal of the patient, the disease diagnosis is employed using the proposed Spizella-based Bi-LSTM Epilepsy seizure prediction technique. Here, the EEG signal is preprocessed to remove noise and artifacts. The pre-processed EEG signal is then used to extract the features, such as the spectral, Hjorth, and statistical features. Then extricated features are integrated to formulate a feature vector, which is then fed to the proposed SBTM model for seizure prediction, in which the Bi-LSTM is tuned using the proposed Spizella optimizer that is designed by fusing the foraging practice of the Spizella by considering the alarm indication and the striving behavior of the Cougar to move towards the next position by considering the alertness to save the Spizella from the snatcher. Here, using the proposed algorithm, the weights of the predictor are tuned to increase the performance of Seizure prediction. Moreover, the normalization-based pre-processing and heart variability-based feature extraction remove the noise from the input and reduce the complexity in terms of computation from the network, respectively. The flow diagram of the proposed SBTM model for epileptic seizure prediction is depicted in Fig. 3.

#### Pre-processing of EEG signal

The EEG acquired from the blockchain comprises noise and artifacts, which can be eliminated using the pre-processing technique. Here, in the proposed method, a bandpass filter^31^ is utilized, in which the signal with the range of 0.05Hz to 75 Hz is filtered to remove the noise.

#### Feature extraction using statistical frequency state features (StFS)

The distinctive features, such as the spectral, Hjorth, and statistical features, are drawn from the pre-processed EEG signal to present the information from the input EEG signal that reduces the computational complexity associated with the predictor. The features, like spectral features, Hjorth features, and statistical features, are concatenated to form the feature vector of the signal that eradicates the computational complexity associated with the raw EEG signal. The concatenated features are given as,

2 $$B = \{ F_{spectral} ,F_{hijorth} ,F_{statistical} \}$$

The feature vector $B$ with dimension$[1 \times 2000]$, which forms the input to the seizure prediction module. The in-depth discussion of the EEG signal-based features is described below.

##### Statistical features

The statistical features summarize the complex datasets into simpler, more manageable forms and help in identifying the patterns within the data. The statistical features, including variance, median, mean, kurtosis, and skewness, and their description is shown in Table 1. Further, the statistical features with the dimension $\left( {N,5} \right)$ extracted from the pre-processed signal are detailed as:

**Table 1:** Statistical Features.

Statistical Features	Expression	Description
Skewness	$Stat_{skew} = \frac{{\sum\limits_{x = 1}^{s} {\left( {U_{x} - \overline{U} } \right)^{3} } }}{{(s - 1)\sigma^{3} }}$	Evaluates the symmetry of the EEG signal
Kurtosis:	$Stat_{kur} = \frac{{U^{4} }}{{\sigma_{stat}^{4} }}$	Evaluates the tailedness of the EEG signal
Mean	$\overline{U} = \sum\limits_{x = 1}^{s} {\frac{{U_{{}} }}{s}}$	Evaluates the average of the EEG signals’ time instances
Median	$Median = \frac{U + 1}{2}$	Provides a robust measure of central tendency in data
Standard deviation	$\sigma = \sqrt {\frac{{(U_{x} - \overline{U} )^{2} }}{s}}$	Evaluates the EEG signal values spread in the distribution

##### Hjorth features

Hjorth descriptor is utilized for measuring the time domain EEG signal’s dynamics, which evaluates the signal complexity. Table 2 shows the description and expression of Hjorth features. In addition, the Hjorth descriptor with the dimension $\left( {N,3} \right)$ comprises three parameters, such as activity, mobility, and complexity. The activity evaluates the strength of the signal, as well as the irregularity of the time function, theoretically. The proportion of the standard deviation of the power spectrum is presented by the mobility parameter. Finally, the complexity parameter indicates the change in frequency evaluated by dividing the standard deviation comparison between the second and first derivative.

**Table 2:** Hjorth Features.

Hjorth Features	Expression	Description
Hjorth Parameter	$J_{0} = \frac{1}{s}\int\limits_{x - 1}^{s} {U^{2} \left( x \right)ds}$	Evaluates the three mental states of the EEG signal
	$$J_{2} = \frac{1}{s}\int\limits_{x - 1}^{s} {\left( {\frac{dU}{{ds}}} \right)^{2} ds}$$	
	$$J_{4} = \frac{1}{s}\int\limits_{x - 1}^{s} {\left( {\frac{{d^{2} U}}{{dx^{2} }}} \right)^{2} dx}$$	
Estimation of Hjorth parameters	$Y^{activity} = J_{0}^{\varsigma }$	Estimation of the above-mentioned Hjorth parameters for the discrete data
	$$Y^{mobility} = \sqrt {\frac{{J_{2}^{\varsigma } }}{{J_{0}^{\varsigma } }}}$$	
	$$Y^{complexity} = \sqrt {\frac{{J_{0}^{\varsigma } }}{{J_{4}^{\varsigma } }} - \frac{{J_{4}^{\varsigma } }}{{J_{0}^{\varsigma } }}}$$	

##### Spectral features

The prediction of Epilepsy seizures based on the smart healthcare scenario uses spectral attributes with the dimension of $\left( {N,5} \right)$ comprising the centroid, spread, kurtosis, skewness, and crest for accurate prediction with minimal computation complexity. The spectral features and their description are shown in Table 3.

**Table 3:** Spectral features.

Spectral features	Expression	Description
Spectral centroid	$Spect_{1} = \sqrt {\frac{{\sum\limits_{{l = j_{1} }}^{{j_{2} }} {d_{g} f_{g} } }}{{\sum\limits_{{l = j_{1} }}^{{a_{j2} }} {f_{g} } }}}$	Evaluates the central or dominant frequency in the EEG signal at a specific moment
Spectral spread	$Spect_{2} = \sqrt {\frac{{\sum\limits_{{l = j_{1} }}^{{j_{2} }} {(d_{b} - Spect_{1} )f_{g} } }}{{\sum\limits_{{l = j_{1} }}^{{j_{2} }} {f_{g} } }}}$	Spectral spread characterizes the frequency components in the EEG signal
Spectral Kurtosis	$Spect_{3} = \sqrt {\frac{{\sum\limits_{{l = j_{1} }}^{{j_{2} }} {(d_{g} - Spect_{1} )^{4} f_{g} } }}{{(Spect_{2} )^{4} \sum\limits_{{l = j_{1} }}^{{a_{j2} }} {f_{g} } }}}$	Spectral kurtosis quantifies the non-periodic nature of the frequency components in an EEG signal
Spectral skewness	$Spect_{4} = \sqrt {\frac{{\sum\limits_{{l = j_{1} }}^{{j_{2} }} {(d_{g} - Spect_{1} )^{3} f_{g} } }}{{(Spect_{2} )^{3} \sum\limits_{{l = j_{1} }}^{{j_{2} }} {f_{g} } }}}$	Distribution of spectral power and potentially detect abnormalities
Spectral crest	$Spect_{5} = \sqrt {\frac{{\max (f_{g} \in \left| {j_{1} ,j_{2} } \right|}}{{\frac{1}{{j_{2} - j_{1} }}\sum\limits_{{l = j_{1} }}^{{a_{j2} }} {f_{g} } }}}$	Evaluates the sharp peaks or spikes in the frequency spectrum of EEG signals

Further, the fused features with the dimension $\left( {N,13} \right)$ extracted from the EEG signals are split into equivalent signals for preserving the structural property of the EEG pattern. Finally, the split signals with the dimension $\left( {N,2000} \right)$ are obtained to highlight the consistent pattern that can be used for the epileptic seizure diagnosis and classification.

#### Proposed SBTM model for epileptic seizure prediction employing EEG features

**Fig. 4:**
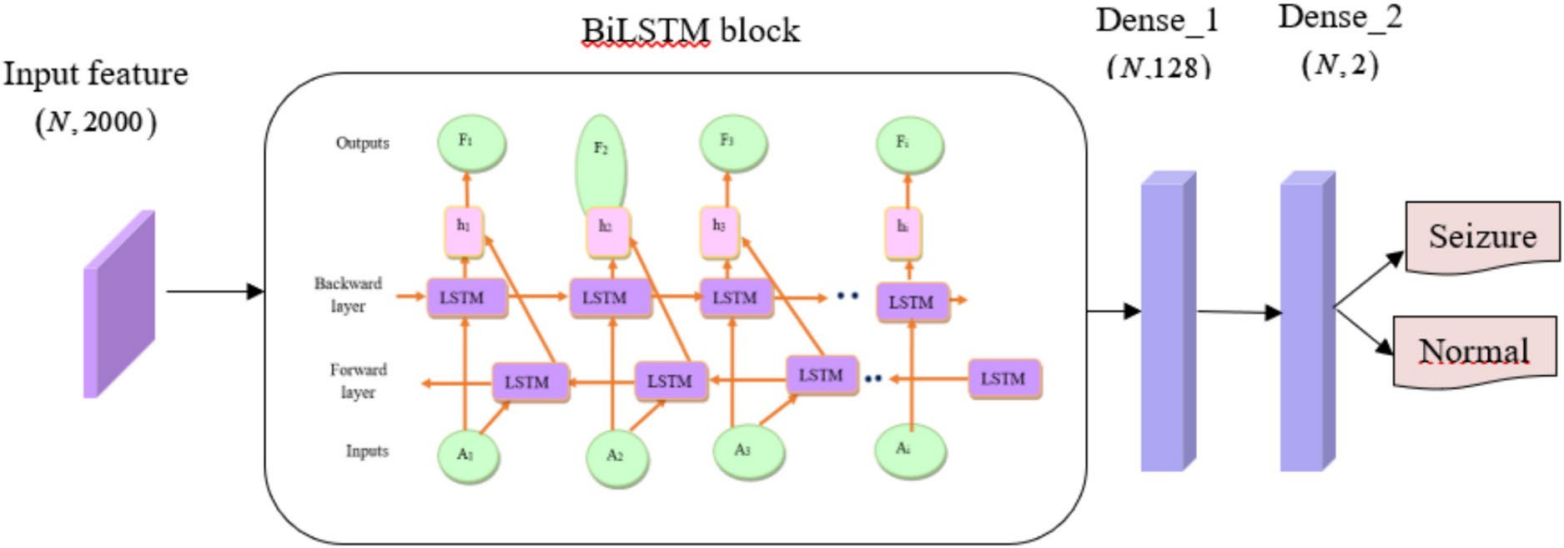
Architecture of the SBTM model.

The SBTM model is utilized for epileptic seizure prediction, for which the newly designed Spizella optimizer is utilized to enhance the performance of the presented disease diagnosis through parameter optimization. The Spizella optimizer substitutes the ADAM optimizer and handles the classifier training with minimal computational complexity, which promotes the prediction accuracy of the model. Moreover, derived features from the signal further minimize the training time, thereby establishing more refined features for prediction, and moreover, the learning from the past and present seizure instances is performed. The overall system stands for the timely diagnosis of the patient. SBTM is a form of recurrent neural network that effectively captures the time-based relationships in sequential data. Specifically, the SBTM analyzes the obtained spatial features and identifies the temporal patterns present in the EEG signal, allowing it to recognize long-term dependencies and contextual information for improved seizure prediction. The features obtained from the feature extraction are utilized for classification or additional analysis to identify the occurrence or non-occurrence of seizures. In summary, the suggested framework provides a thorough method for examining EEG signals and identifying epileptic seizures, utilizing the strengths of deep learning models to enhance the precision and effectiveness of seizure detection in medical environments. The architecture of the SBTM model comprises the input layer, a BiLSTM layer, dense layers, and a softmax layer. In addition, the SBTM layer constitutes the output, input, and hidden layers with additional forward and backward layers with peculiar performance. The architecture of the SBTM model used for epileptic seizure prediction is depicted in Fig. 4.

The input layer receives input sequences, typically represented as vectors. The input layer serves as a channel through which sequential data flows in the forward and backward directions in the subsequent SBTM layer. The SBTM consists of output, input, and forget gate, and is notated as, $A_{i} ,D_{i} \,and\,H_{i}$ respectively, with the bias and weight indicated as $C$ and $E$ , respectively. The point multiplication and the element-wise multiplication are noted as $*$ , respectively.

*Input gate:* The input gate ($A_{i}$) is responsible for controlling the information flow into the cell state. This decides the relevant information from the current input $\left( {B_{i} } \right)$ and previous hidden state $\left( {h_{i - 1} } \right)$ to be stored using the sigmoid function, resulting values between 0 and 1. The information of the current state is updated and is formulated as,

3 $$A_{i} = sigmoid\left( {E_{a} \cdot \left[ {h_{i - 1} ,B_{i} } \right] + C_{A} } \right)$$

*Forget gate:* The forget gate ($D_{i}$) considers the current input $\left( {B_{i} } \right)$ and the previous state information ($h_{i - 1}$) for storing the historical information by discarding or retaining the information using the sigmoid function, and is referred to as,

4 $$D_{i} = sigmoid\left( {E_{D} \cdot \left[ {h_{i - 1} ,B_{i} } \right] + C_{D} } \right)$$

*Cell state:* It considers the new cell state $F_{i - 1}$ and the candidate value $\overline{{F_{i} }}$ along with the $D_{i}$ and $A_{i}$, which potentially carries the information through the network, where the output of the memory cell $F_{i}$ can be expressed as,

5 $$\overline{{F_{i} }} = \tanh \left( {E_{F} \cdot \left[ {h_{i - 1} ,B_{i} } \right] + C_{F} } \right)$$

6 $$F_{i} = D_{i} * F_{i - 1} + A_{i} * \overline{{F_{i} }}$$

*Output gate:* The status of the memory cell is controlled by this gate, which determines the information to be outputted as the hidden state. The output gate is denoted as $H_{i}$ and is formulated as,

7 $$H_{i} = sigmoid\left( {E_{H} \cdot \left[ {h_{i - 1} ,B_{i} } \right] + C_{H} } \right)$$

Thus, the output evaluated by the LSTM in the current hidden state $\left( {h_{i} } \right)$ is expressed as,

8 $$h_{i} = H_{i} * \tanh \left( {F_{i} } \right)$$

The SBTM for the prediction of the epileptic seizure by considering the backward ($\mathop {h_{i} }\limits^{ \leftarrow }$) and the forward ($\mathop {h_{i} }\limits^{ \to }$) direction can be expressed as,

9 $$\mathop {h_{i} }\limits^{ \to } = LSTM\left( {B_{i} ,\mathop {h_{i - 1} }\limits^{ \to } } \right)$$

10 $$\mathop {h_{i} }\limits^{ \leftarrow } = LSTM\left( {B_{i} ,\mathop {h_{i - 1} }\limits^{ \leftarrow } } \right)$$

11 $$G_{i} = \left[ {\mathop {h_{i} }\limits^{ \to } ,\mathop {h_{i} }\limits^{ \leftarrow } } \right]$$

Here, the signal is encoded from the beginning to the end by the forward layer and the reverse direction by the backward layer for better feature extraction. Thus, the above-mentioned weights are tuned using the proposed Spizella optimizer to increase the accuracy of the model through the global best solution. The outcome from the SBTM layer is fed to the fully connected layer. This layer maps the output from the SBTM layer to high dimensional space. This enables the model to learn more complex patterns of the data.

Further, he output from the fully connected layer is fed to the softmax layer, which transforms the output into probability distributions across multiple classes. Finally, the output layer takes the generated probability from the softmax layer and makes the final prediction. Table 4 presents the layer details of the SBTM.

**Table 4:** Layer details of SBTM.

Type of layer	Shape of the output
Sequence Input	(None, 2000)
BiLSTM	100 hidden units
Fully Connected	(None, 2)
Softmax	(None, 2)
Classification Output	(None, 2)

##### Proposed spizella optimizer

Spizella optimization is designed to tune the hyperparameters of the SBTM model for improving the detection accuracy. Specifically, the spizella optimizer is developed by hybridizing the food-searching practice of the Sparrows by considering the alarm indication^32^ and the striving behavior of the cougar to move towards the next position by considering the alertness^33^. These behaviors potentially save the search solution from being stuck in local optimal solutions thereby attaining the global best solution to adjust the hyperparameters. According to the substantial characteristics of Spizella optimization algorithm, it possibly resolves convergence problems and reduce exhibiting false positives during evaluation, making robust for epileptic seizure prediction using the SBTM model.

*Initialization:* In the Spizella optimization, the solutions are randomly generated based on the hyperparameters such as weights and biases of the SBTM model as $M = \left[ {C,E} \right]$. Further, the Spizella’s are located in the search space, in which the number of Spizella is notated as $e$, and the dimension is notated as $k$_,_ and they are expressed as,

12 $$M = \left[ {\begin{array}{*{20}c} {M_{1,1} } & {M_{1,2} } & \cdots & \cdots & {M_{1,k} } \\ {M_{2,1} } & {M_{2,2} } & \cdots & \cdots & {M_{2,k} } \\ \vdots & \vdots & \vdots & \vdots & \vdots \\ {M_{e,1} } & {M_{e,2} } & \cdots & \cdots & {M_{e,k} } \\ \end{array} } \right]$$

*Fitness:* The fitness is evaluated as in Eq. (29), and the Spizella with the best fitness is considered as the best producer.

13 $$Fitness = \frac{{\left( {M_{accuracy} + M_{sensitivity} + M_{specificity} } \right)}}{3}$$

where the terms are accuracy, sensitivity, and specificity, respectively, which are defined in Eqs. (39), (38), and (37), respectively.

*Exploration Phase* ($for\,i = 1:NP$*): The exploitation phase* occurs while the search agent has the highest probability of finding the food location and guides the borrowers to obtain the food to enhance their energy. Here $NP$ indicates the number of producers in the entire population. As per assumptions (1) and (2), by considering the producer, Spizella’s location can be formulated as,

14 $$M_{u,v}^{i + 1} = \left\{ \begin{gathered} M_{u,v}^{i} .\exp \left( {\frac{ - u}{{\gamma .i_{\max } }}} \right)\quad ,\quad if\;T2 < R \hfill \\ M_{u,v} + N.O\quad ,\quad \quad \quad \;if\;T2 \ge R \hfill \\ \end{gathered} \right.$$

Here, the random number $N$ that follows the normal distribution, the alarm signal is indicated by $T2$ a range between [0,1] for which the safety threshold $R$ ranges $[0.5,1]$. The maximum iteration is notated as $i_{\max }$ and the random number $\gamma$ ranges between [0,1] and the matrix with the dimension $[1 \times k]$ is denoted as $O$. In addition, when $T2 < R$ it is considered that the attackers are not available, in contrast, when $T2 \ge R$ it is indicated that the attacker is available. In this condition, the Cougar’s striving and discovering behavior is considered due to its high alertness. A threshold is considered to identify behavior, in which the Cougar moves with better velocity in the discovering phase, looks around the area within the search space, and moves toward the next safer location. Thus, the updated equation of the Cougar is expressed as,

15 $$M_{u,v}^{i + 1} = M_{u,v}^{{}} + Vel_{u,v}^{{}}$$

where the parameter $Vel_{u,v}^{{}}$ refers to the velocity of the Cougar while discovering the new area, and it can be expressed as,

16 $$Vel_{u,v}^{{}} = Vel_{u,v}^{{}} + q1 \times p1 \times \left( {M_{best,\,v}^{{}} - M_{u,v}^{{}} } \right)$$

where the random number ranges between [0,1] is indicated as $q1$ and $p1$ is the constant. The best location of the Cougar is denoted as $M_{best,\,v}^{{}}$. As per the rule in^23^ for the hybridization of the characteristic behaviors, the position updation can be expressed as,

17 $$M_{u,v}^{i + 1} = 0.5\left[ {M_{u,v}^{i + 1} } \right]_{spizella} + 0.5\left[ {M_{u,v}^{i + 1} } \right]_{Cougar}$$

18 $$M_{u,v}^{i + 1} = 0.5\left[ {M_{u,v} + N.O} \right] + 0.5\left[ {M_{u,v}^{{}} + Vel_{u,v}^{{}} } \right]$$

19 $$M_{u,v}^{i + 1} = 0.5\left[ {M_{u,v} + N.O} \right] + 0.5\left[ {M_{u,v}^{{}} + \left( {Vel_{u,v}^{{}} + q1 \times p1 \times \left( {M_{best,\,v}^{{}} - M_{u,v}^{{}} } \right)} \right)} \right]$$

Thus, hybridizing the discovering phase of the Cougar with the alarming capability of the Spizella aids in maintaining the diversification and intensification phase in search of a global best solution. The high alertness of the Spizella helps to escape from the attacker, and hence, the population can be maintained, which leads to safer exploration of more search areas to attain the best solution.

*Exploitation Phase* ($if\,i = 1:N$): The borrower’s position is updated by taking the assumptions (4) and (5). The borrowers follow the producers for the enhancement of energy, and the producer moves to the next location if the food is not available in the current location. The update equation can be expressed as,

20 $$M_{u,v}^{i + 1} = \left\{ \begin{gathered} N.\exp \left( {\frac{{M_{bad}^{i} - M_{u,v}^{i} }}{{\gamma .i_{\max } }}} \right)\quad ,\quad \quad \quad \quad \quad if\;u > e/2 \hfill \\ M_{P}^{i + 1} + \left| {M_{u,v}^{i} - M_{P}^{i + 1} } \right|.Q^{ + } .S\quad ,\quad \quad \quad \;otherwise \hfill \\ \end{gathered} \right.$$

where the worst location of the spizella is denoted as $M_{bad}$ , and the producer’s position based on the optimal solution is indicated as $M_{P}$. The assumption $u > e/2$ depicts the borrower is in a starving condition and requires food for survival with the lowest fitness. Besides, 10% to 20% of the spizella is considered in danger, and the position updation based on assumption 6 can be expressed as,

21 $$M_{u,v}^{i + 1} = \left\{ \begin{gathered} M_{good}^{i} + \gamma \left| {M_{u,v}^{i\tau } - M_{good}^{i} } \right|,\quad \quad \quad \quad \quad \quad if\;m_{u} > m_{n} \hfill \\ M_{u,v}^{i} + X\left( {\frac{{\left| {M_{u,v}^{i} - M_{bad}^{i} } \right|}}{{(m_{u} - m_{w} ) + \alpha }}} \right)\quad ,\quad \quad \quad \;if\quad m_{u} = m_{n} \hfill \\ \end{gathered} \right.$$

**Fig. 5:**
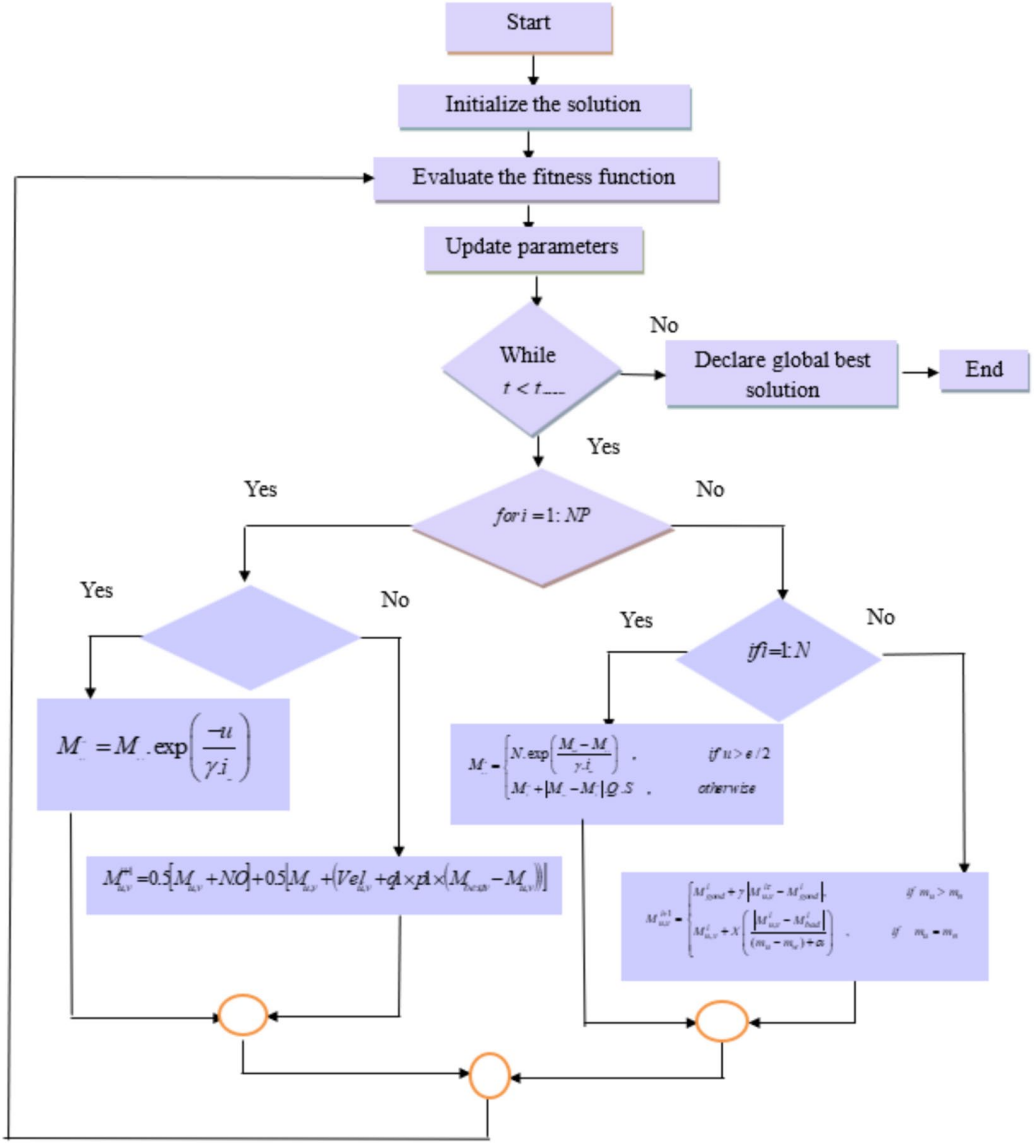
Flowchart of the Spizella optimizer.

Here, the parameter $\gamma$ is utilized to control the step size, and it ranges among $[0,1]$, the best solution is denoted as $M_{good}^{i}$, and the random number is notated as $X \in [ - 1,1]$. The worst and the best fitness values are represented as $m_{w}$ and $m_{n}$ respectively. The Spizella at the middle of the search space is denoted as $m_{u} = m_{n}$, and the direction to move towards the safer position is denoted as $X$. *Termination:* The aforementioned procedure is repeated until the attainment of the global best solution or the maximum number of iterations. The flowchart of the Spizella algorithm is presented in Fig. 5, given below.

## Result and discussion

In this section, the results obtained with the implementation of the proposed smart healthcare-based Epilepsy seizure prediction is discussed in detail.

### Experimental setup

The proposed smart healthcare-based Epilepsy seizure prediction is implemented using the MATLAB tool running in the Windows 11 Operating system with 16GB RAM and 0.34ms processing time. The hyperparameters of the proposed model and the optimization are provided in Tables 5 and 6 below.

**Table 5:** Hyperparameters of the model.

Hyperparameters	Ranges/types
Learning rate	0.001
Embedding size	300
Batch size	128
Number of epochs	100
Number of hidden layers	2
Number of neurons in each hidden layer	128, 64
Activation function	ReLU
Regularization	L2
Dropout rate	0.5
Regularization parameter	0.001
Optimizer	Adam
Loss function	Cross entropy

**Table 6:** Hyperparameters of the optimization.

Hyperparameters	Ranges/types
Population size	100
Number of generations	100
Crossover probability	0.8
Mutation probability	0.1
Selection method	Roulette Wheel
Crossover method	One-point
Mutation method	Gaussian

### Experimental results

**Fig. 6:**
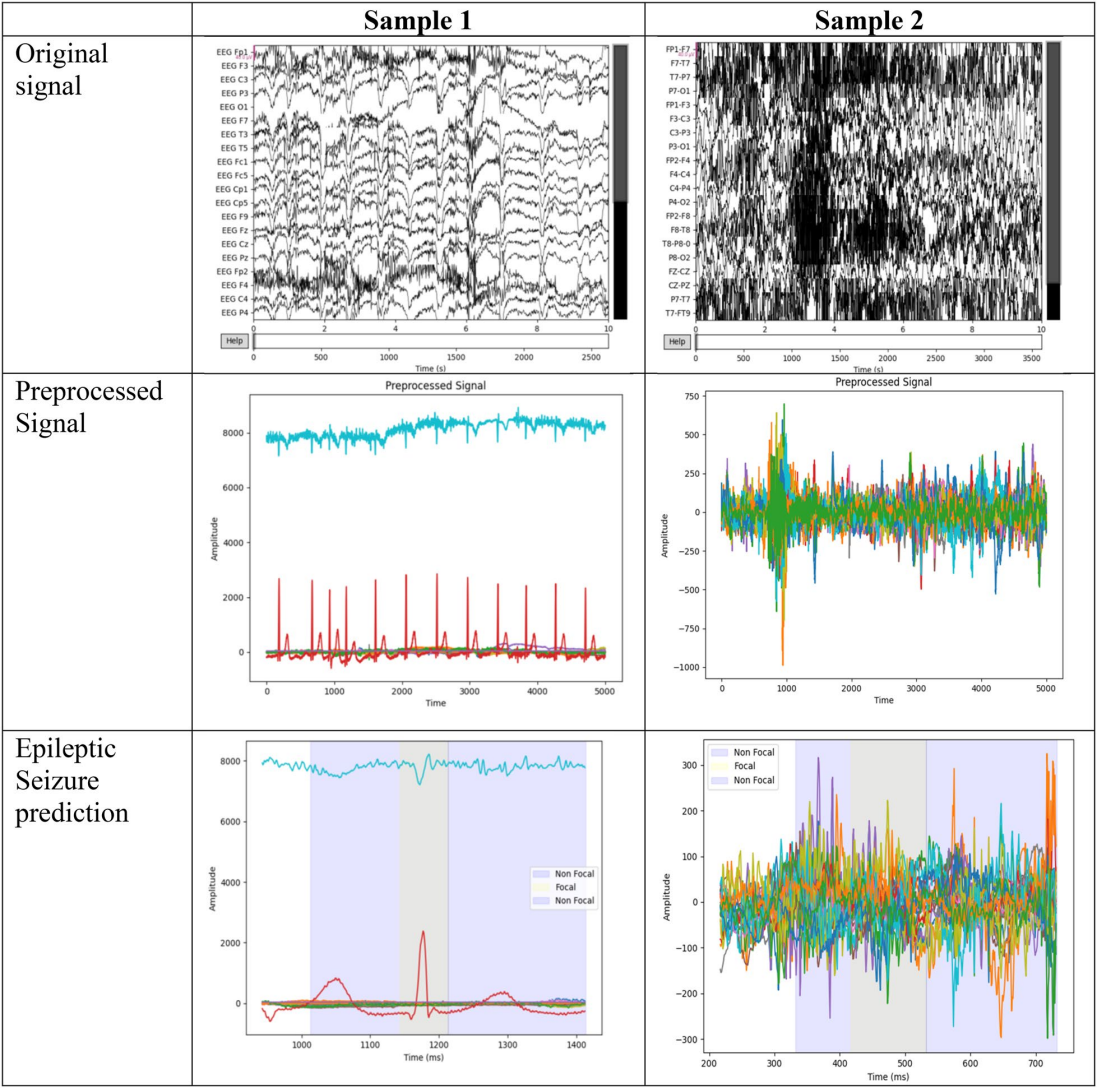
Experimental results obtained using the SBTM Model.

The experimental results obtained with the proposed IoT-assisted epileptic seizure prediction using the SBTM model are shown in Fig. 6. Initially, the input sample EEG signals used for the epileptic seizure prediction, followed by the preprocessed signal using the bandpass filter, and the final epileptic seizure prediction output is obtained with SBTM model.

### Dataset description

**Fig. 7:**
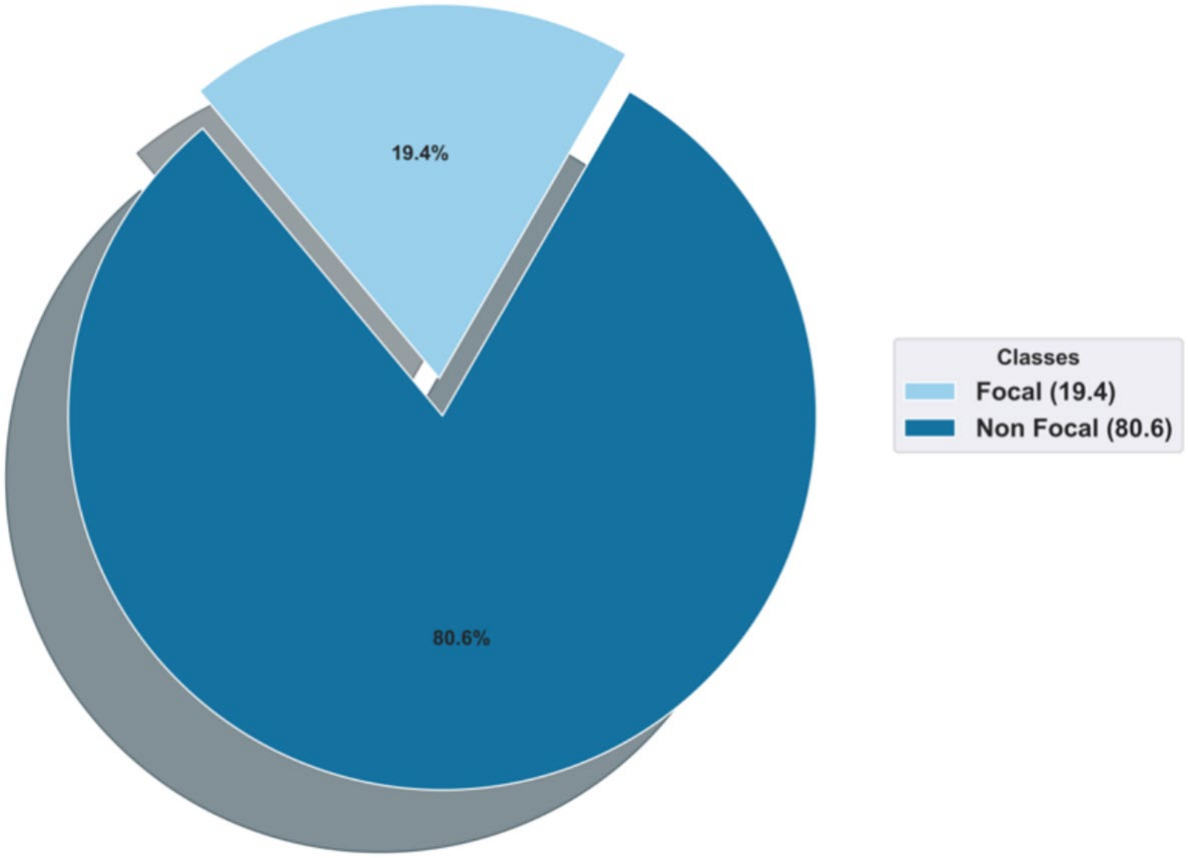
Visualization of CHB-MIT dataset.

The dataset used to assess the effectiveness of the developed Spizella-based Bi-LSTM for disease prediction is done through the CHB-MIT dataset^34^. The dataset is collected from the Children’s Hospital Boston, which consists of EEG recordings from young patients who had uncontrollable seizures. Following the discontinuation of anti-seizure medication, the subjects were observed for a few days at most to assess their suitability for surgical intervention and to define their seizures. 22 subjects’ recordings (five males, ages 3–22, and seventeen females, ages 1.5–19) were compiled into 23 cases. Every signal was sampled with 16 bits of resolution and 256 samples per second. Figure 7 shows the proportion of the Focal and Non Focal classes in the CHB-MIT dataset. The majority of files have 23 EEG signals (sometimes 24 or 26). In the CHB-MIT dataset, there exist the focal and non-focal classes, accommodating about 19.4% and 80.6% respectively. For assessing generalizability, the research utilizes real-time dataset for external testing on this experiment.

### Performance metrics

The metrics used to assess the developed seizure prediction method are specificity, sensitivity, and accuracy described in Table 7, as follows:

**Table 7:** Performance Metrics.

Metrics	Expression	Description
Accuracy	$Accuracy = \frac{TP + TN}{{TP + TN + FP + FN}}$	Evaluation of the proposed disease prediction either the correctly predicted true or false case
Sensitivity	$Sensitivity = \frac{{TP_{t} }}{{TP + FN_{f} }}$	Evaluates the correctly predicted patients with epileptic seizures
Specificity	$Specificity = \frac{TN}{{TN_{f} + FP}}$	The exactness of the prediction is evaluated through the specificity
F1-score	$F1 - score = \frac{2 \times precision \times recall}{{precision + recall}}$	Evaluate the harmonic mean of precision and recall

### Performance analysis

This section describes the performance analysis of SBTM model in terms of varying training percentage and K-fold values.

#### Performance analysis based on training percentage

**Fig. 8:**
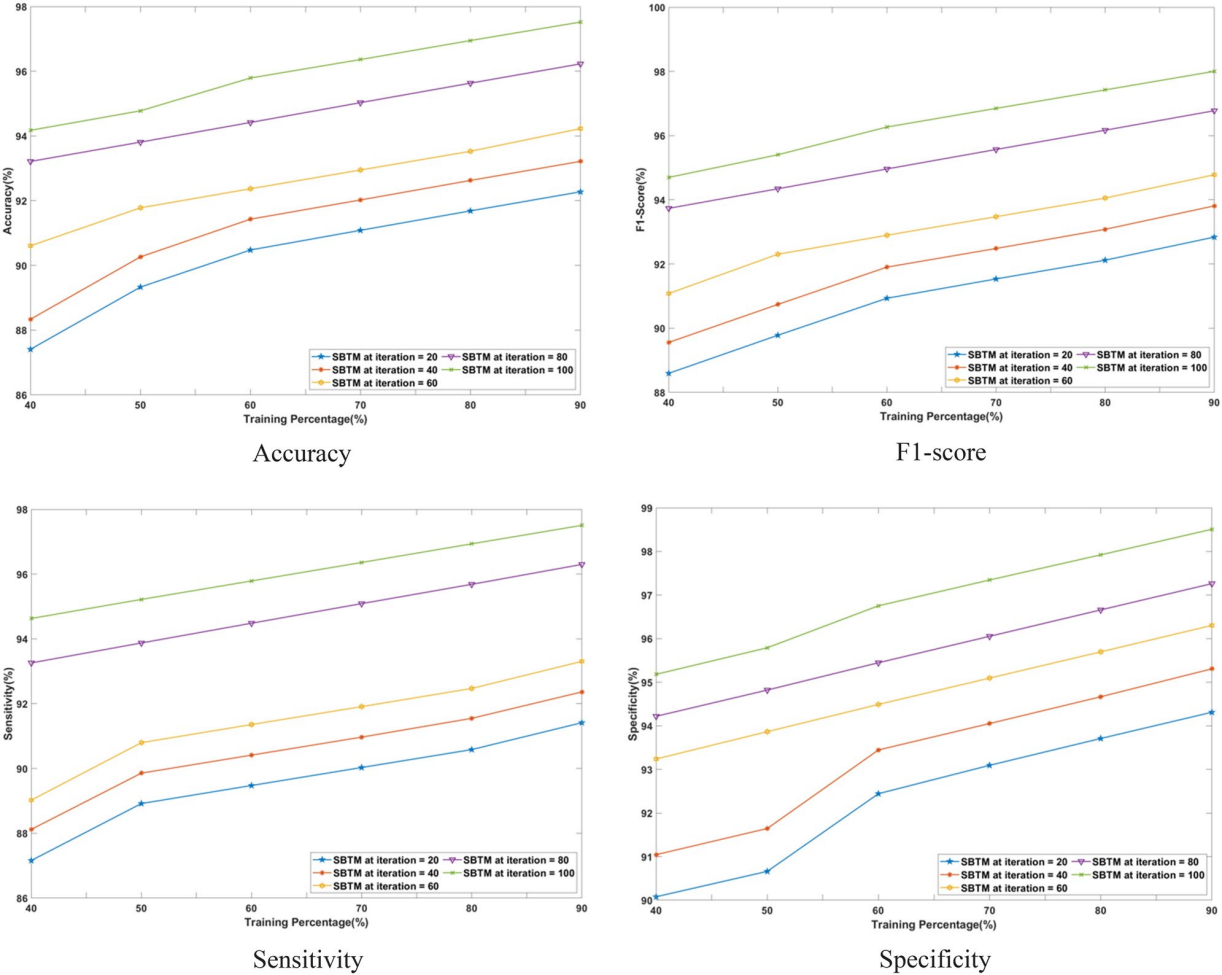
Performance analysis with Training percentage on CHB-MIT Scalp EEG database.

**Fig. 9:**
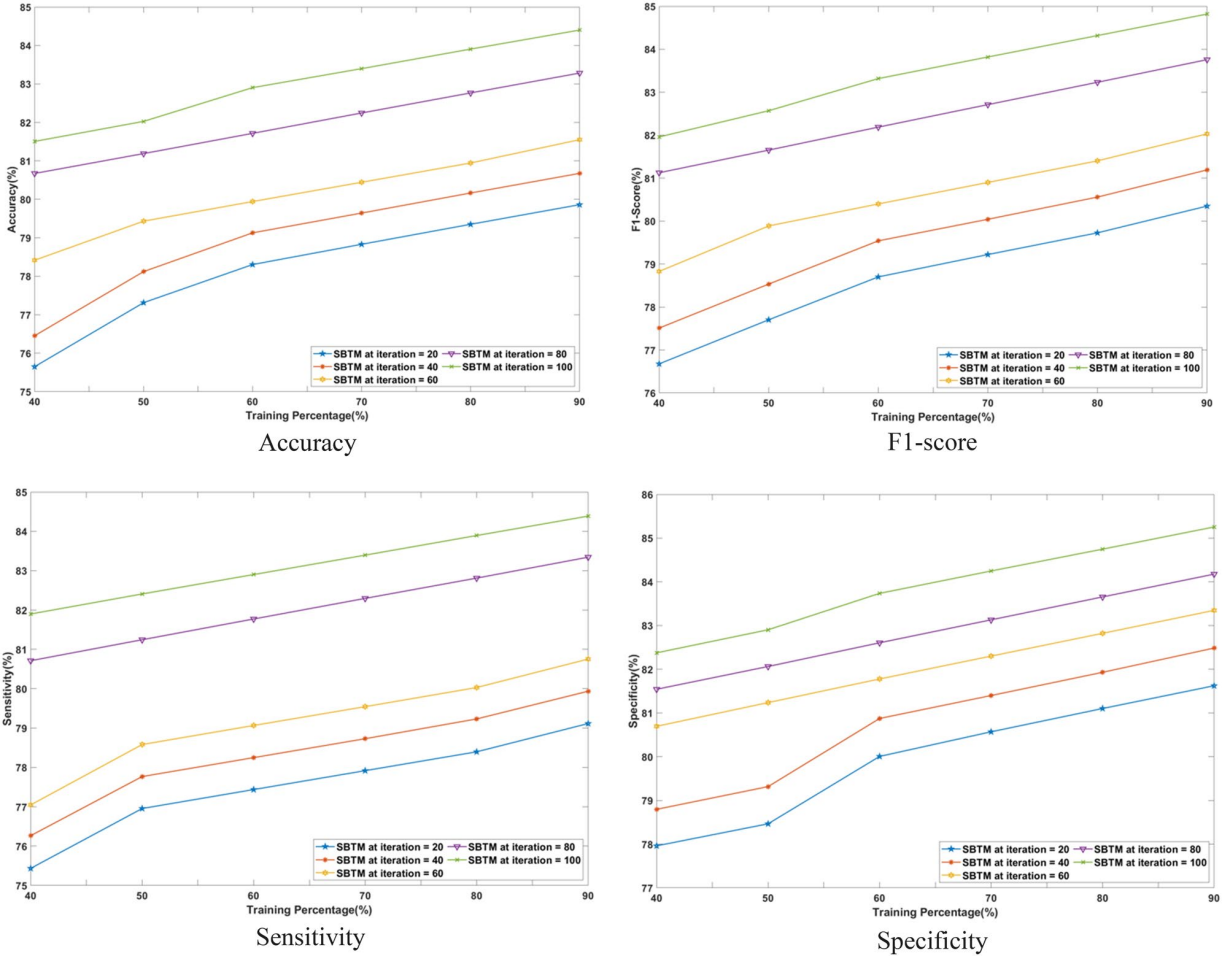
Performance analysis with Training percentage on Real-time dataset.

The superiority of the SBTM model evaluated in terms of varying the training percentages from 40 to 90% is discussed in this section. The performance is analyzed for a range of iterations from 20 to 100 epochs using CHB-MIT Scalp EEG and Real-time dataset as represented in Figs. 8 and 9, respectively.

Using the CHB-MIT Scalp EEG database for epochs 20, the SBTM model attains an accuracy of 92.28%, which is further improved to 97.52% for 100 epochs. Using a real-time dataset for 90% training, the accuracy of SBTM is measured as 79.86% and 84.40% for 20 epochs and 100 epochs, respectively, indicating the capability of the model in learning the training data. Moreover, SBTM gains F1-scores of 97.74% and 84.81% at iteration 100, using CHB-MIT Scalp EEG and real-time dataset, respectively. Furthermore, using the CHB-MIT Scalp EEG database, the sensitivities of 91.41% and 97.50% are evaluated at iterations 20 and 100, correspondingly. Meanwhile, using the real-time dataset, maximum sensitivity values of 79.12% and 84.39% are obtained at iterations 20 and 100. Furthermore, the SBTM gains specificities of 98.50% and 85.26% at iteration 100 for the CHB-MIT Scalp EEG and real-time dataset, respectively. The higher sensitivity indicates that the SBTM minimizes the false alarm rates through exhibiting a deeper understanding on seizure occurrence.

#### Performance analysis based on K-fold

**Fig. 10:**
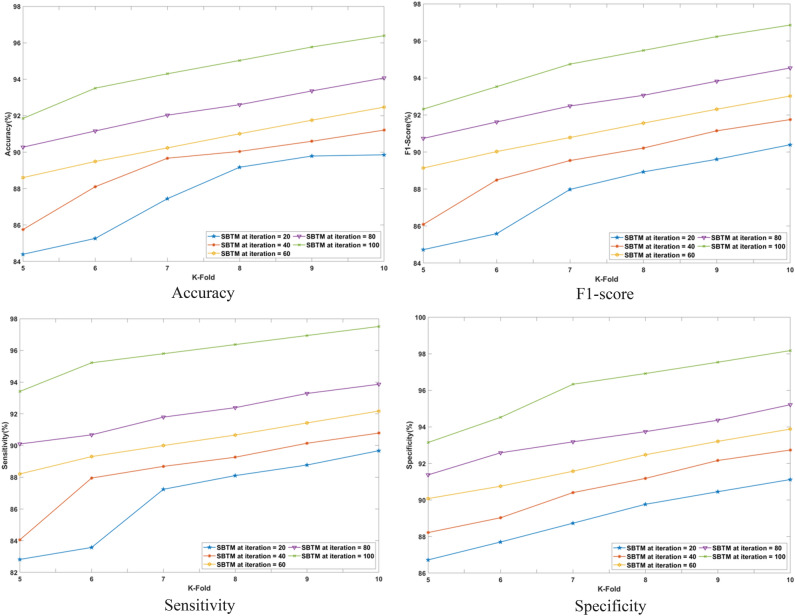
Performance analysis with K-fold validation on CHB-MIT Scalp EEG dataset.

**Fig. 11:**
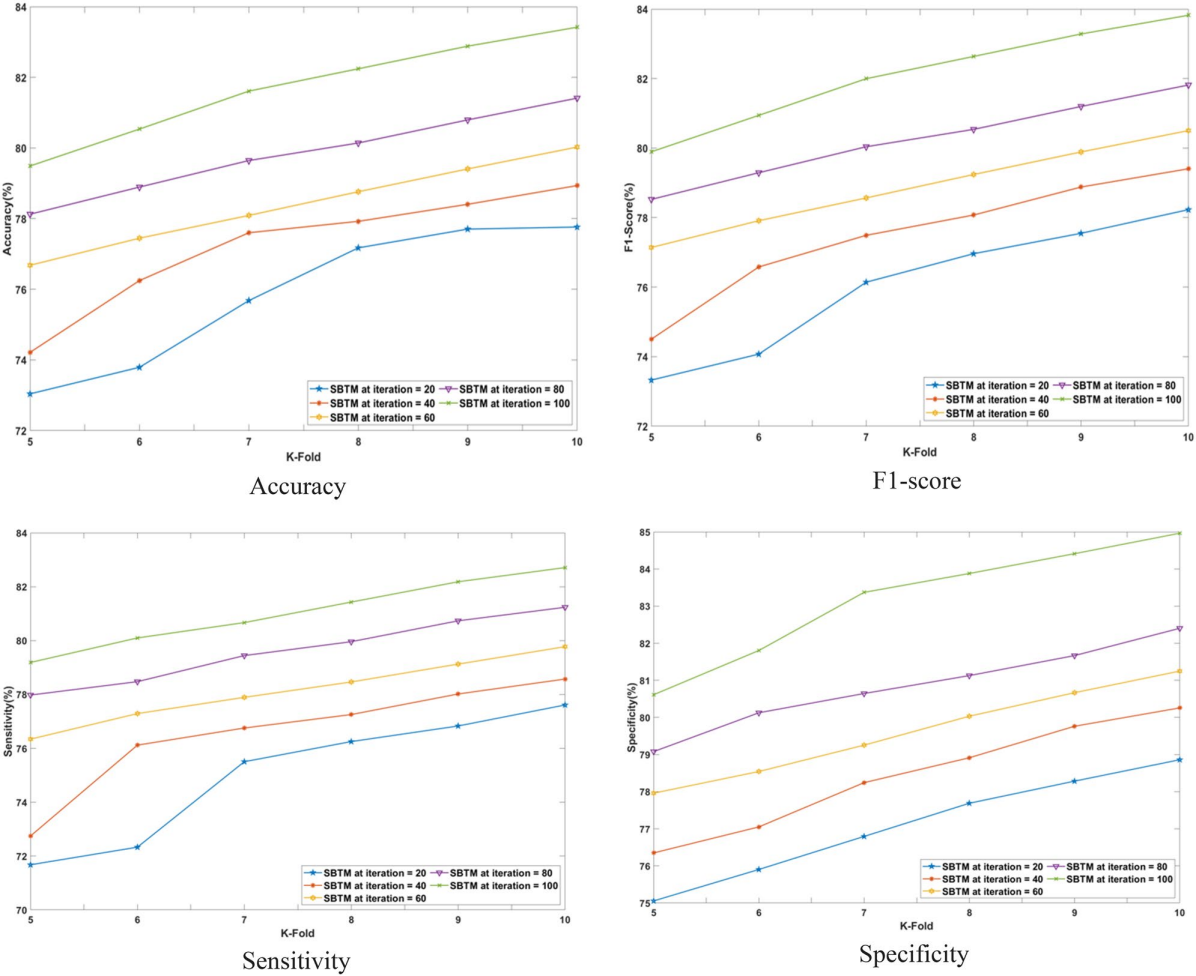
Performance analysis with K-fold validation on real-time dataset.

The performance of the SBTM model evaluated in terms of varying the K-folds from 10 to 11 is discussed in this section. The performance is analyzed for a range of iterations from 20 to 100 epochs using CHB-MIT Scalp EEG and real-time dataset as represented in Figs. 10 and 11, respectively. Using the CHB-MIT Scalp EEG dataset, SBTM gains F1-scores of 90.39% and 96.85% at iterations 20 and 100, respectively. For the real-time dataset, the SBTM model showcases F1-scores of 78.23% and 83.82%, correspondingly for iterations 20 and 100. Using the CHB-MIT Scalp EEG database for epochs 20, the SBTM model attains an accuracy of 89.85% that is further improved to 96.39% for 100 epochs. Using real-time dataset for K-fold 10, the accuracies of SBTM is 77.76% for 20 epochs and 83.42% for 100 epochs, respectively, indicating the accurate prediction of epileptic seizures. Furthermore, for K-fold 10, the SBTM gains specificities of 98.17% and 84.96% at iteration 100 using the CHB-MIT Scalp EEG and real-time dataset, respectively, indicating the lower false positives. The SBTM achieves 89.67% and 97.51% sensitivities at iterations 20 and 100, correspondingly using the CHB-MIT Scalp EEG dataset. Meanwhile, using the real-time dataset, maximum sensitivity values of 77.61% and 84.39% are obtained at iterations 20 and 100 for K-fold 10.

### Comparative analysis

The evaluation is carried out to showcase the effectiveness of the SBTM model over existing techniques. For comparison, techniques used to assess the proposed method are RF^19^, PSO-ANN^20^, ForeSeiz^18^, SCFHSAN^21^, MLF-CNN^22^, DCNN^17^, GTDA-KNN^27^, CL-ATBiLSTM^26^, and DEF-DSVM^28^. Further, the comparative analysis of the proposed approach in terms of k-fold and training percentage analysis is discussed in the section below.

#### Analysis based on training percentage

**Fig. 12:**
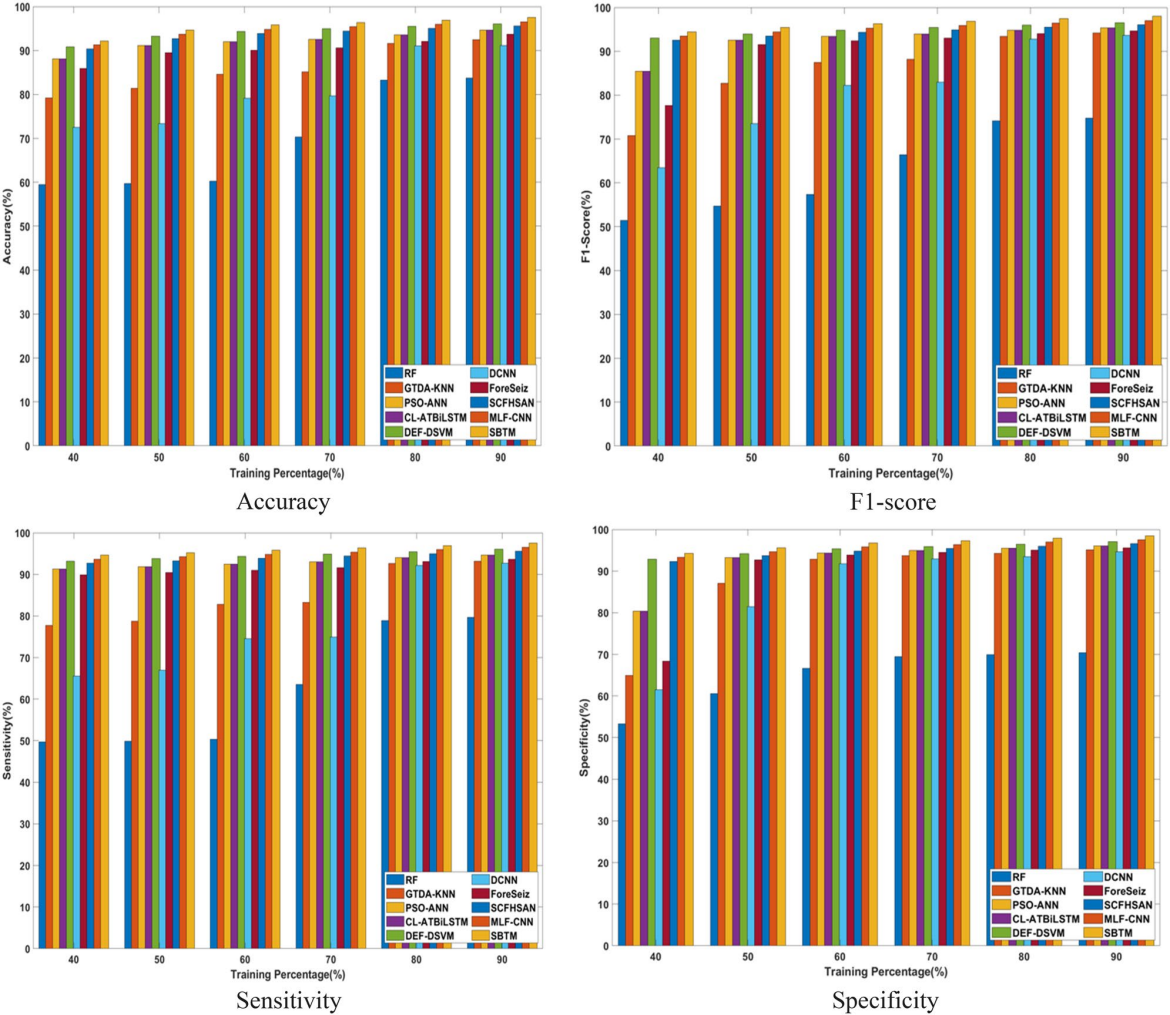
Comparative analysis based on training percentage using CHB-MIT Scalp EEG dataset.

**Fig. 13:**
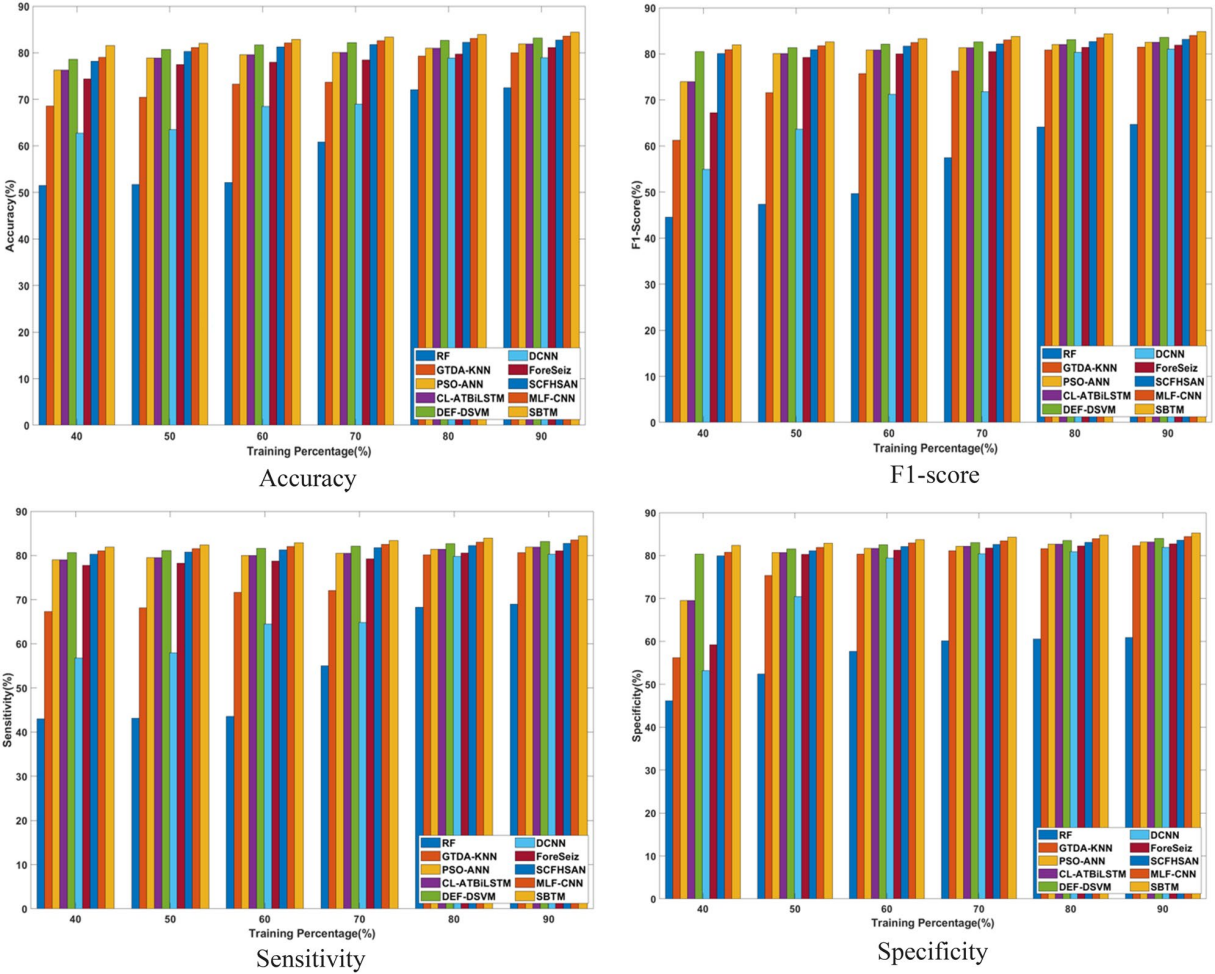
Comparative analysis based on training percentage using real-time dataset.

The comparison of the SBTM model with the conventional methods using the CHB-MIT Scalp EEG dataset and the real-time dataset in terms of varying training percentage is presented in Figs. 12 and 13. Using the CHB-MIT Scalp EEG dataset, the SBTM model showcases 97.52% for 90% training, which shows a relative improvement of 1.49% over DEF-DSVM, 2.95% over CL-ATBiLSTM, and 14.10% over RF. For the real-time dataset, the SBTM model achieves 84.40% accuracy, improved over PSO-ANN, DEF-DSVM, and SCFHSAN by 2.95%, 1.49%, and 1.98% respectively. The highly accurate performance of SBTM enables precise and on-time interventions achieved using the adaptive weight tuning of the Spizella algorithm. Subsequently, the sensitivity achieved by the SBTM model using CHB-MIT Scalp EEG dataset is 97.51% when utilizing 90% of the training data, representing a significant improvement of 18.24% against RF, 2.96% against PSO-ANN, 4.91% against DCNN, appropriately. Similarly, for the real-time dataset, the SBTM model achieves 84.39% sensitivity for 90% of training, which is improved over CL-ATBiLSTM by 2.96%, DCNN by 4.91%, and SCFHSAN by 1.99%. Similarly, the specificity attained by the SBTM CHB-MIT Scalp EEG database is 98.51% when trained with 90% of the data, demonstrating the relative improvement of 28.58%, 2.45%, 3.91% over the existing techniques RF, PSO-ANN, and DCNN, respectively. Moreover, for real-time dataset, the SBTM demonstrates 85.26% specificity with improvement of 1.47% against DCNN, 2.45% against PSO-ANN, and 28.58% against RF, correspondingly. Furthermore, the F1-score of SBTM is 98% and 84.82%, showcasing higher improvement of 0.99% against MLF-CNN using both datasets. The results indicates that SBTM model demonstrates superior performance in epileptic seizure prediction against the existing techniques. The inclusion of the improved Bi-LSTM in the proposed model assisted in capturing the temporal patterns and nonlinear dynamics of EEG signals, resulting in the effective prediction of epileptic seizures. Additionally, the Spizella Optimization optimally tunes the hyperparameters of the SBTM model, leading to enhanced prediction accuracy.

#### Analysis based on K-fold

**Fig. 14:**
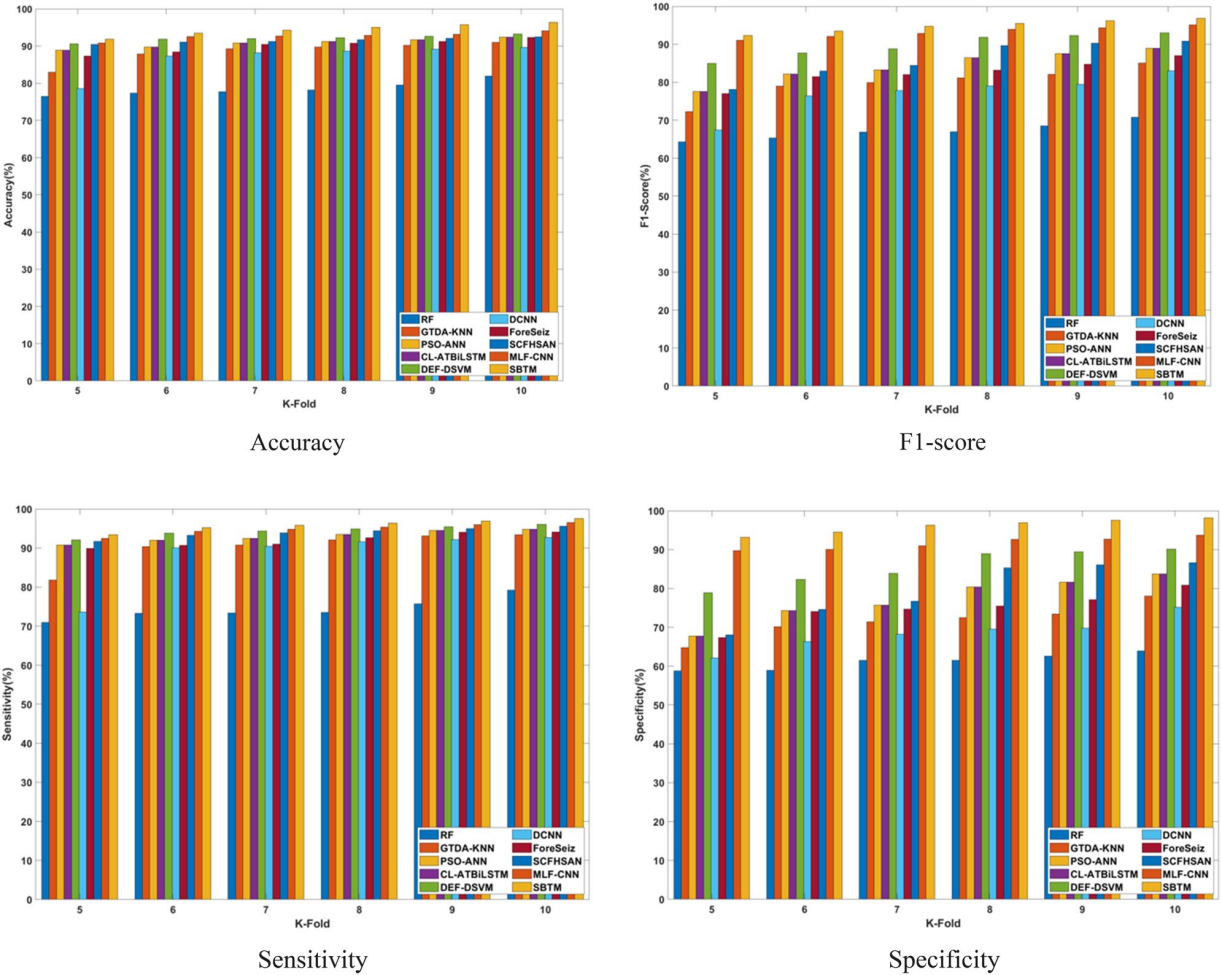
Comparative analysis based on k-fold using CHB-MIT Scalp EEG dataset.

**Fig. 15:**
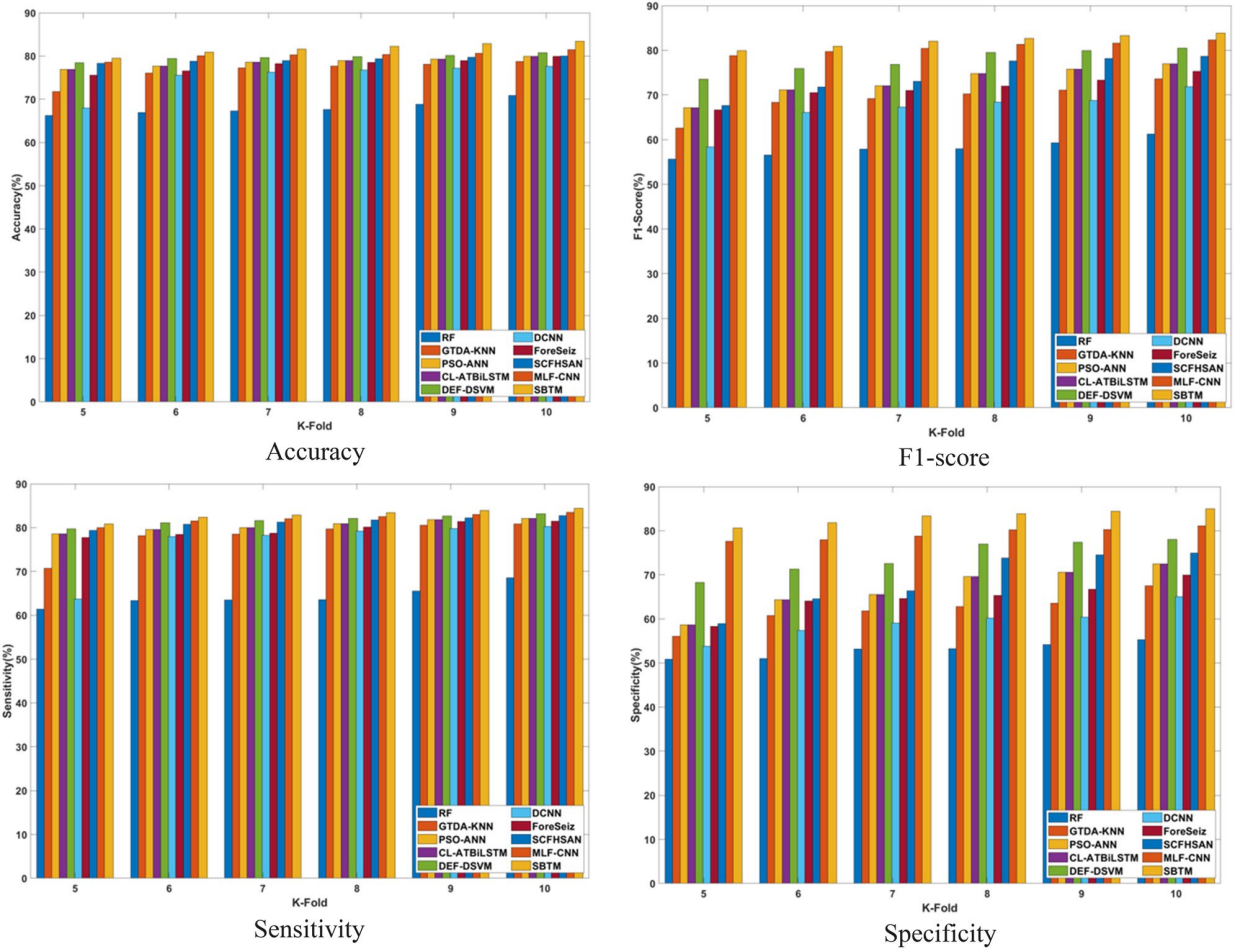
Comparative analysis based on k-fold using real-time dataset.

The comparison of the SBTM model with the conventional methods using CHB-MIT Scalp EEG dataset and real-time dataset in terms of varying K-folds is presented in Figs. 14 and 15. The specificity attained by the SBTM using CHB-MIT Scalp EEG database is 98.17% when trained with K-fold 10, demonstrating the relative improvement of 34.89%, 14.71%, 23.41% over the existing techniques such as RF, PSO-ANN, and DCNN, respectively. Moreover, for real-time dataset, the SBTM demonstrates 84.96% specificity with improvement of 23.41% against DCNN, 20.52% against GTDA-KNN, and 14.71% against CL-ATBiLSTM, correspondingly. Using the CHB-MIT Scalp EEG dataset, the SBTM model showcases 96.39% accuracy for 90% training which shows a relative improvement of 3.21% over DEF-DSVM, 4.15% over CL-ATBiLSTM, and 15.01% over RF. For the real-time dataset, the SBTM model achieves 83.42% accuracy improved than the PSO-ANN, DEF-DSVM, and SCFHSAN by 4.15%, 3.22%, and 4.09% respectively. The highly accurate performance of SBTM enables precise and on-time interventions achieved using the adaptive weight tuning of Spizella algorithm. Furthermore, the F1-score of SBTM is 96.85% and 83.82% using the two datasets, showcasing the improvement over DEF-DSVM by 3.96% and 3.96%, respectively. Subsequently, the sensitivity achieved by the SBTM model using CHB-MIT Scalp EEG dataset is 97.51% when utilizing 90% of the training data, representing a significant improvement of 18.24% against RF, 2.96% against PSO-ANN, 4.91% against DCNN, appropriately. Similarly, for the real-time dataset, the SBTM model achieves 84.39% sensitivity for 90% training that is improved over CL-ATBiLSTM by 2.74%, DCNN by 4.91%, and SCFHSAN by 1.99% correspondingly. From the overall analysis, the proposed SBTM model outperformed the other existing techniques prevalent in epileptic seizure prediction. More specifically, the SBTM model, combining the strength of the BiLSTM architecture and Spizella optimization, offers high learning efficiency and captures the dynamic dependencies between different channels of EEG.

### Comparative discussion

In the proposed research, the SBTM model is compared with other existing methods, such as RF, PSO-ANN, DCNN, ForeSeiz, SCFHSAN, and MLF-CNN, to demonstrate the efficiency of the proposed model. Compared to the above existing methods, the presented SBTM model offers superior diagnosis performance. In contrast, existing techniques, including the RF, PSO-ANN, DCNN, ForeSeiz, SCFHSAN, and MLF-CNN, are observed with inherent limitations. For instance, RF faces difficulty in analyzing the high-dimensional data and is found with slower inference times due to the large number of trees. In addition, the PSO-ANN converges prematurely to a suboptimal solution, particularly in noisy environments, and is prone to overfitting. Meanwhile, the MLF-CNN model lacks better generalization performance with the relatively small sample size and requires a large number of training samples to achieve better performance. However, the proposed SBTM model addressed the existing challenges via the application of the Spizella optimization and optimized SBTM Model, in which the improved SBTM architecture excels in capturing the temporal dependencies and nonlinear dynamics associated with the EEG signals, making the model highly efficient for predicting the epileptic seizure patterns. Furthermore, the Spizella optimization adaptively fine-tunes the SBTM model, resulting in enhanced performance. By validating on additional real-time dataset, we are confident that the model has learned robust, generalizable patterns across the patients’ records. Tables 8 and 9 depict the comparative discussion of the proposed method and other baseline techniques obtained in terms of two datasets, including the CHB-MIT Scalp EEG dataset and real-time dataset.

**Table 8:** Comparative Discussion using CHB-MIT Scalp EEG dataset.

Methods/ Metrics	TP-90				K-fold 10			
	Accuracy (%)	Sensitivity (%)	F1-score (%)	Specificity (%)	Accuracy (%)	Sensitivity (%)	Specificity (%)	F1-score (%)
RF^19^	83.77	79.72	74.75	70.36	81.92	79.24	63.91	70.76
GTDA-KNN^27^	92.44	93.2	94.16	95.14	90.99	93.40	78.03	85.03
PSO-ANN^20^	94.64	94.62	95.35	96.10	92.39	94.83	83.74	88.94
CL-ATBiLSTM^26^	94.64	94.62	95.35	96.1	92.39	94.83	83.74	88.94
DEF-DSVM^28^	96.07	96.05	96.55	97.06	93.29	96.04	90.17	93.02
DCNN^17^	91.18	92.72	93.68	94.66	89.65	92.72	75.19	83.04
ForeSeiz^18^	93.69	93.67	94.63	95.62	92.33	94.10	80.87	86.98
SCFHSAN^21^	95.60	95.57	96.07	96.58	92.45	95.57	86.61	90.87
MLF-CNN^22^	96.55	96.52	97.03	97.54	94.13	96.52	93.74	95.11
SBTM	97.52	97.51	98	98.51	95.11	97.51	94.73	96.10

**Table 9:** Comparative Discussion using real-time dataset.

Methods/ Metrics	TP-90				K-fold 10			
	Accuracy (%)	Sensitivity (%)	F1-score (%)	Specificity (%)	Accuracy (%)	Sensitivity (%)	Specificity (%)	F1-score (%)
RF^19^	72.50	68.99	64.69	60.89	70.90	68.58	55.31	61.24
GTDA-KNN^27^	80.00	80.66	81.49	82.34	78.75	80.84	67.53	73.59
PSO-ANN^20^	81.91	81.89	82.52	83.17	79.96	82.08	72.47	76.97
CL-ATBiLSTM^26^	81.91	81.89	82.52	83.17	79.96	82.08	72.47	76.97
DEF-DSVM^28^	83.14	83.12	83.56	84.00	80.74	83.12	78.04	80.50
DCNN^17^	78.91	80.25	81.07	81.92	77.59	80.25	65.07	71.87
ForeSeiz^18^	81.09	81.07	81.90	82.75	79.91	81.44	69.99	75.28
SCFHSAN^21^	82.73	82.71	83.15	83.58	80.01	82.71	74.95	78.64
MLF-CNN^22^	83.56	83.53	83.97	84.41	81.46	83.53	81.12	82.31
SBTM	84.40	84.39	84.82	85.26	83.42	84.39	84.96	83.82

### Confusion matrix

**Fig. 16:**
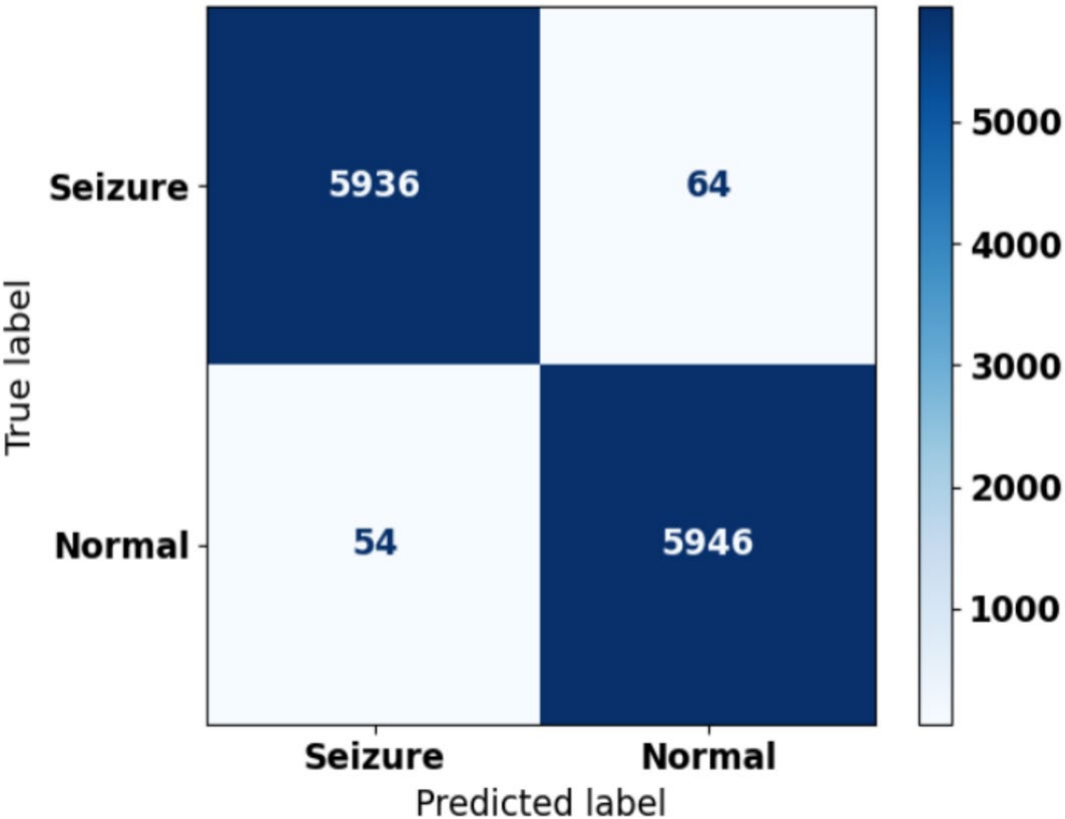
Confusion Matrix.

The confusion matrix of the proposed SBTM model for the epileptic seizure prediction is shown in Fig. 16. Specifically, a confusion matrix assists in assessing the performance as a key indicator of the classification model via contrasting the predicted labels with the true labels that exist in the database. From the confusion matrix using the CHB-MIT dataset, it is identified that which SBTM model predicted the 5936 seizure instances and 5946 normal instances correctly. The proposed model correctly predicted seizure instances with few misdetections. Furthermore, the matrix reveals the efficiency of the suggested model in epileptic seizure prediction.

### Convergence analysis

**Fig. 17:**
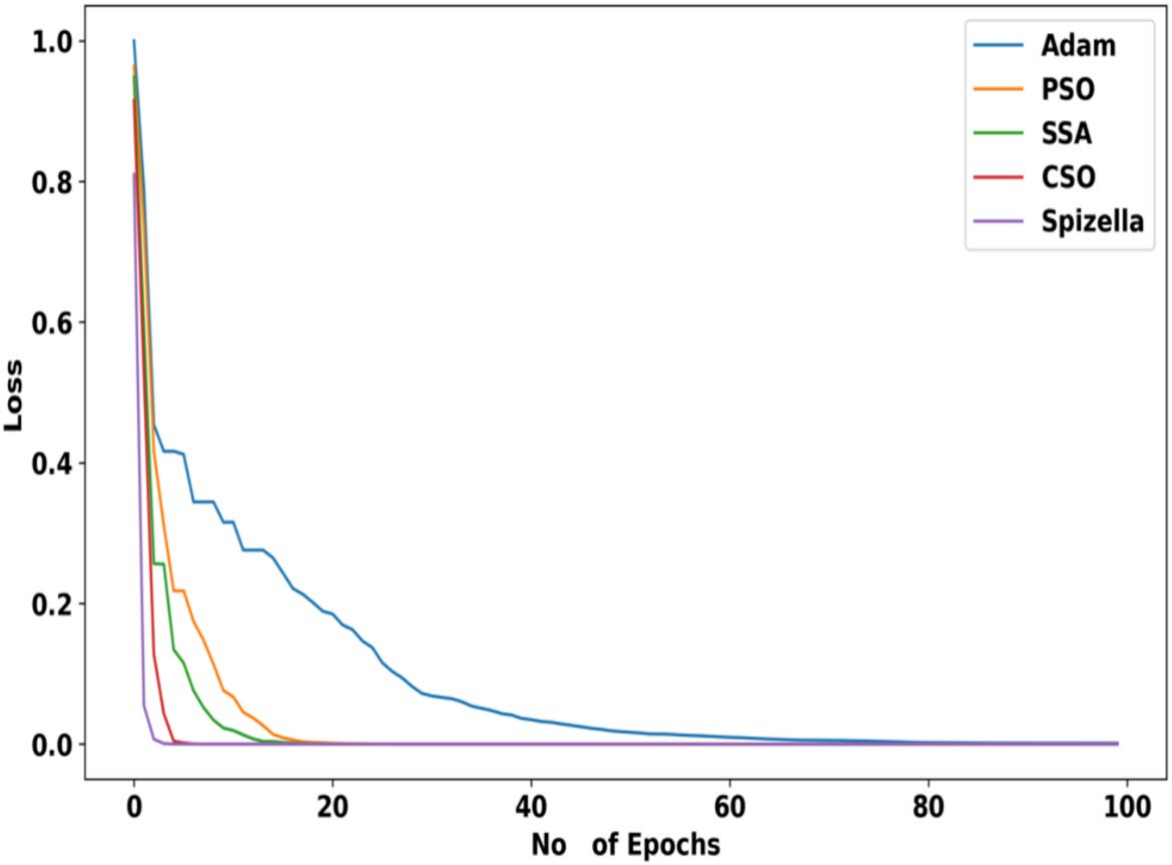
Convergence Analysis.

The convergence analysis of the presented SBTM model with existing optimization algorithms is depicted in Fig. 17. From the convergence analysis, the Spizella approach attained a very low loss 0.81 for epoch 1, which is minimum compared with other optimization algorithms including the Adam, PSO, SSA, and CSO. However, these existing algorithms had attained 1, 0.94, 0.95, and 0.91 loss respectively. Based on the observation, this graph eventually stabilizes a consistent value, indicating an optimal solution has been found for parameter tuning. Upon specification, the incorporation of the unique traits in the Spizella algorithm offers an effective global search resulting in the optimal solution, and also avoids the risk of being trapped in local optima. Consequently, the proposed SBTM model attained faster convergence and achieved minimal loss values, resulting in an effective training process.

### Training time analysis

**Fig. 18:**
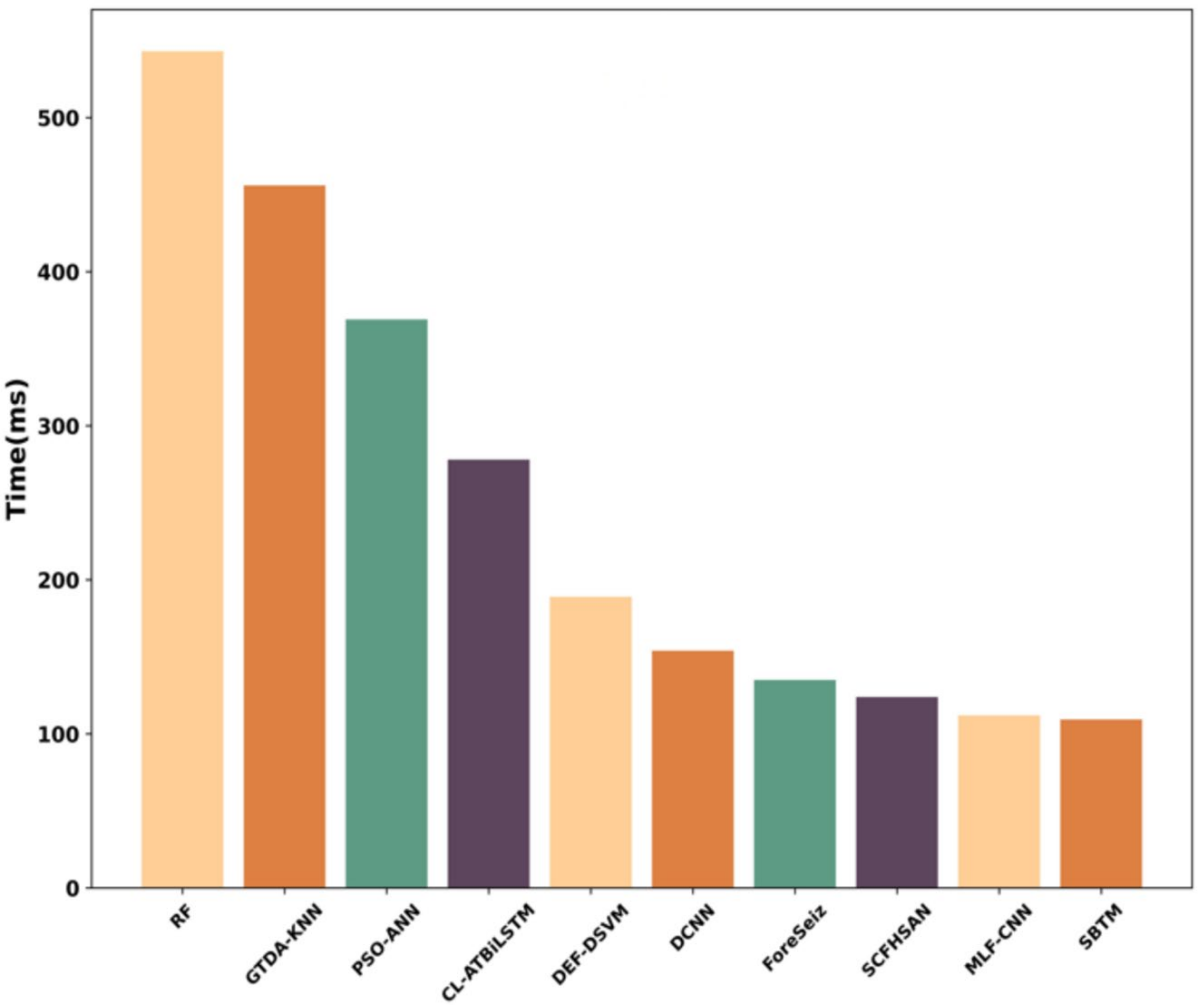
Training Time Analysis.

Figure 18 depicts the training time analysis, which is measured in milliseconds (ms), and validating the model’s complexity as well as computational resources. According to the results, the proposed model only requires 109.34ms for computation, while the existing such as RF, GTDA-KNN, PSO-ANN, CL-ATBiLSTM, DEF-DSVM, DCNN, ForeSeiz, SCFHSAN, and MLF-CNN methods require more training time, reporting 543ms, 456ms, 369ms, 278ms, 189ms, 154ms, 135ms, 124ms, and 112ms, respectively. As the proposed SBTM model integrates the Spizella optimization algorithm for hyperparameter tuning, which reduces the model’s complexity while computing. In addition to that, the lightweight nature of the SBTM simplifies the pipeline compared to multi-step traditional approaches, thereby revealing robustness.

### Receiver operating characteristic curve analysis

**Fig. 19:**
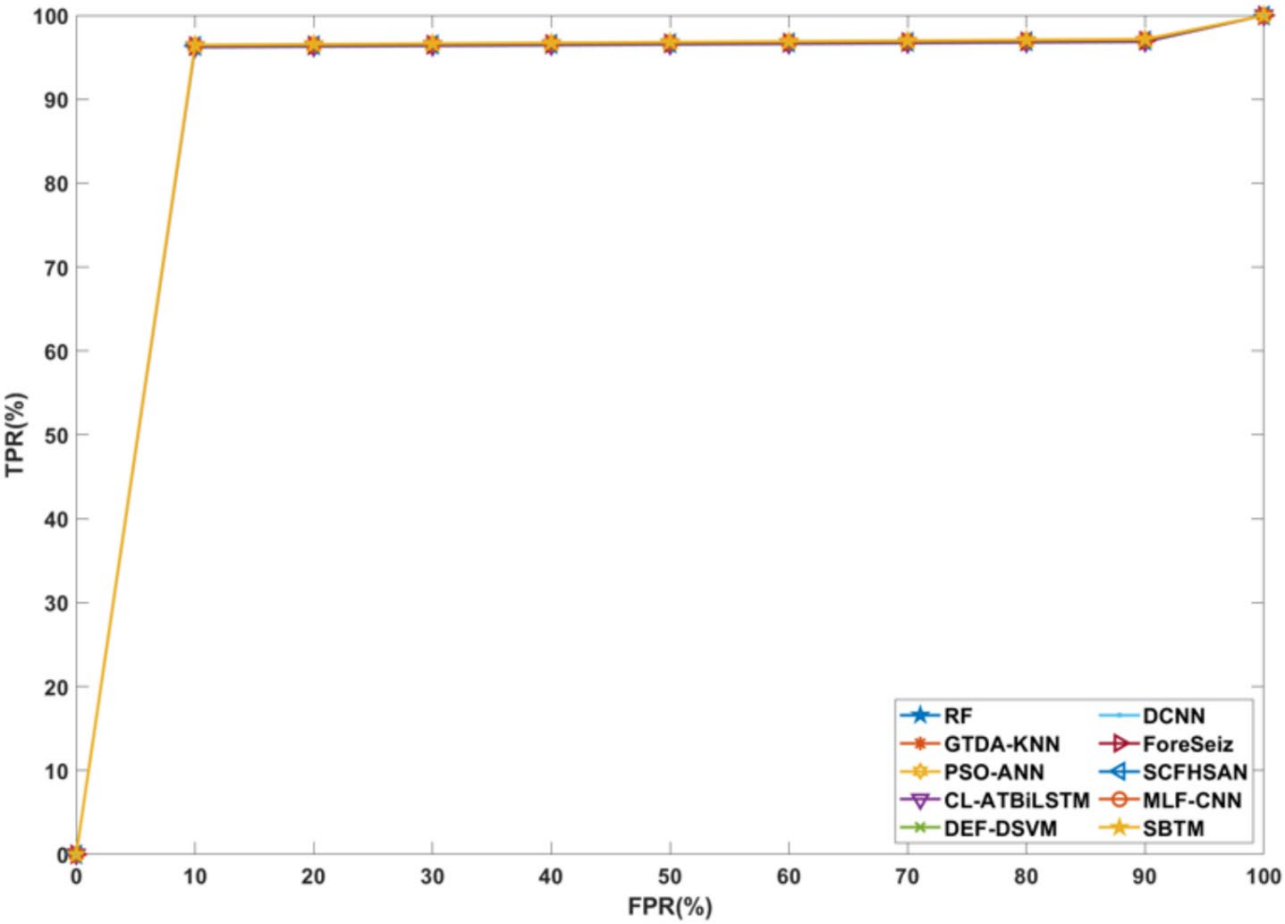
ROC curve.

Figure 19 visualizes the Receiver Operating Characteristic (ROC) curve, in which the performance of the models is plotted as true positive rate on the y-axis and false positive rate on the x-axis for different thresholds. From the analysis, the proposed classifier correctly distinguishes between seizures and non-seizures compared to other methods. Based on this analysis, the SBTM model is capable of performing with an imbalanced dataset and is highly reliable for real-time applications.

### Ablation study

**Fig. 20:**
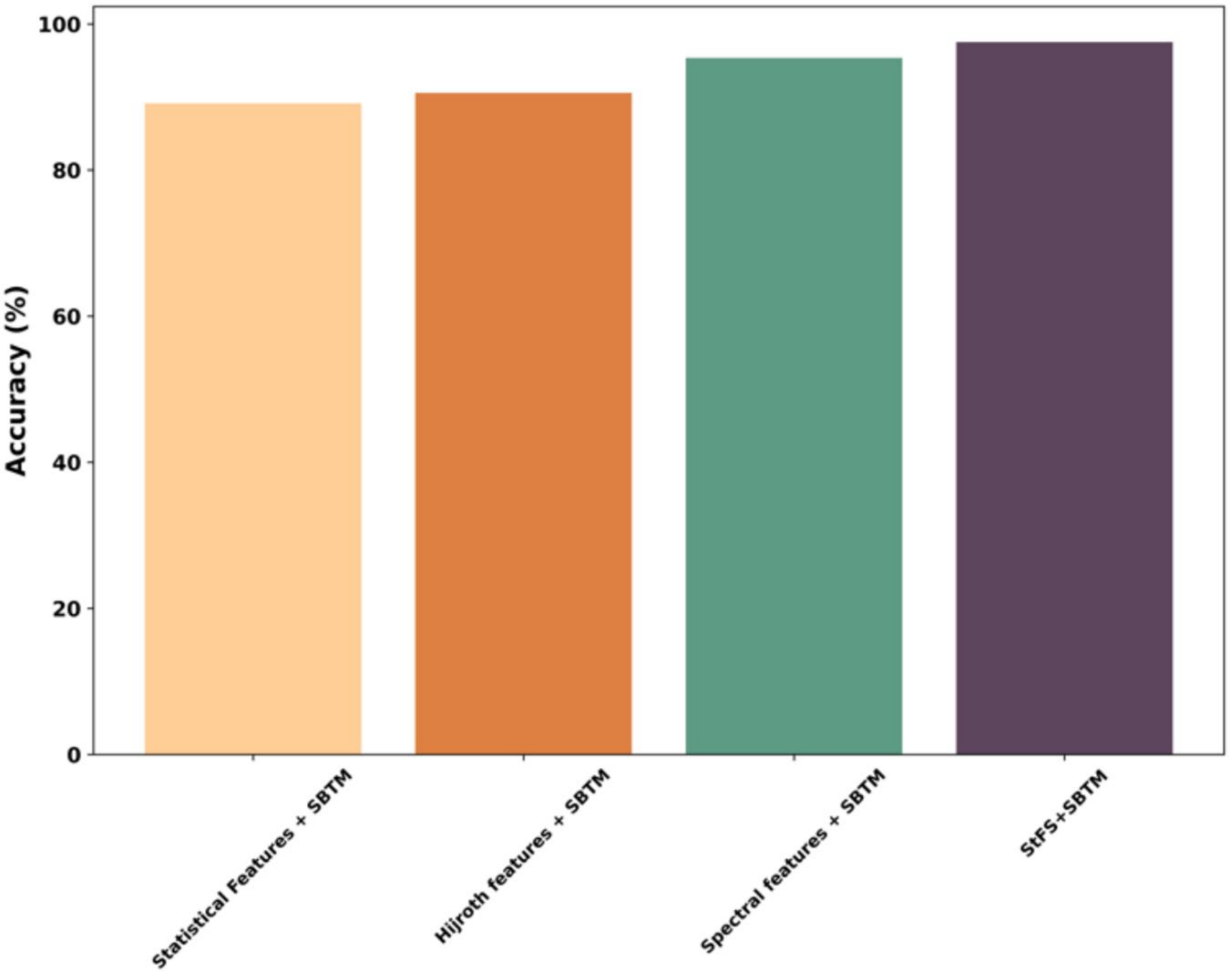
Ablation study.

Ablation study of the proposed method based on different features is shown in Fig. 20, which evaluates the contribution of each component, such as statistical, Hjorth, spectral features, and the StFS, for epileptic seizure detection using the metric accuracy. According to the evaluation, the SBTM gains 97.52% accuracy when combining StFS feature extraction mechanism, while others have shown minimal performance. Based on this analysis, the SBTM model is effective at capturing deep, spectral features using StFS, which are quite significant and optimal for better learning and training the model. Such consequences minimize computational complexity and enhance detection results.

### Security analysis

**Fig. 21:**
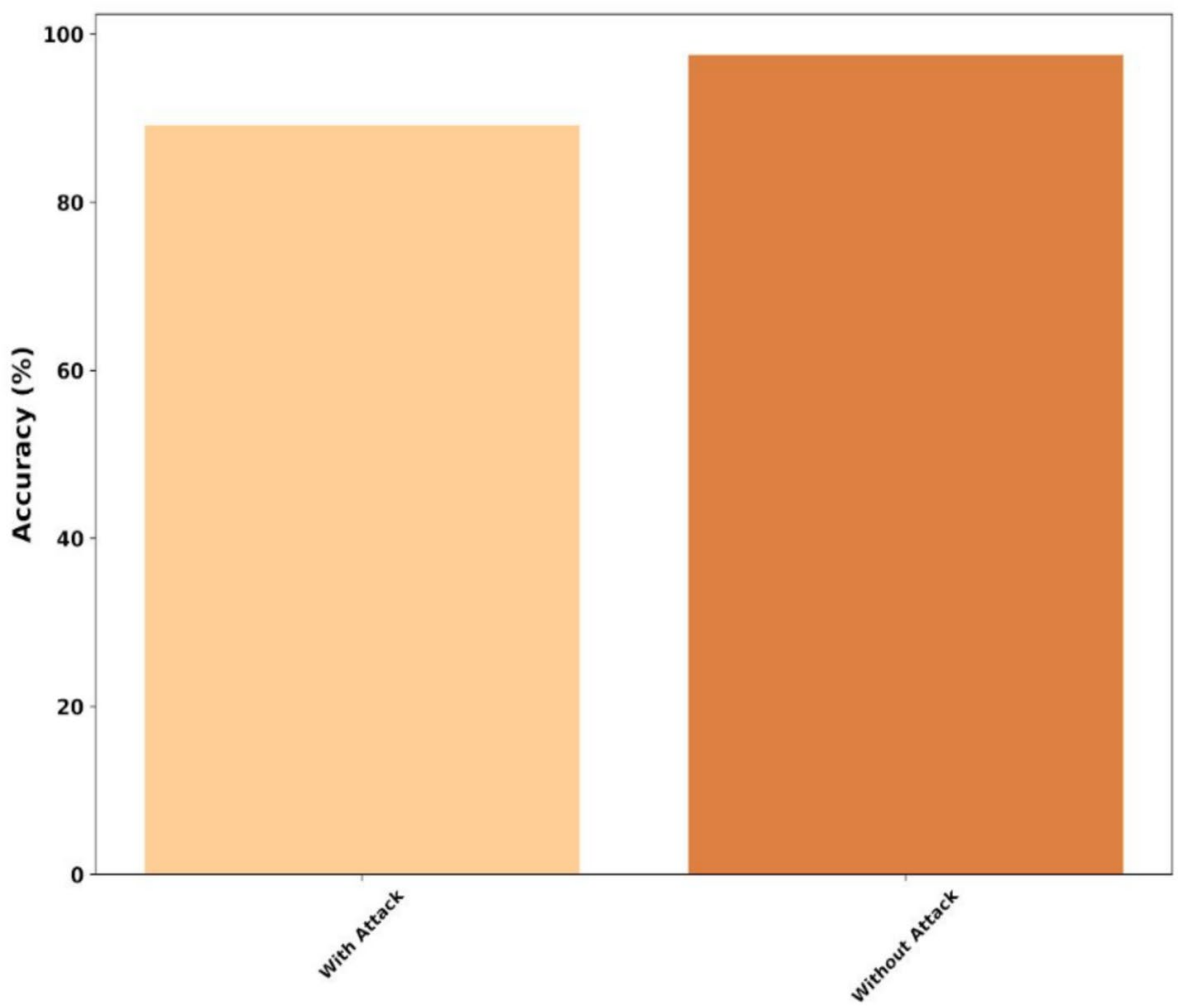
Security analysis.

Figure 21 depicts the security analysis of the proposed model. As the research includes blockchain for EEG storage, the security analysis is conducted with and without attack basis. With attack, the SBTM model reported a low accuracy of 89.12%, and for the scenario of without attack, the SBTM model exhibited a high accuracy of 97.52%. Based on the security analysis, the proposed model shows quite average performance under attacks, but claimed maximum performance under no attack scenario.

### Statistical analysis

Statistical analysis is carried out to evaluate the robustness of the results obtained with respect to metrics like accuracy, sensitivity, and specificity with statistical parameters, namely mean, best, and variance. Tables 10 and 11 depict the statistical analysis carried out for the proposed method and existing methods with 90% of training using the CHB-MIT dataset and the real-time dataset. From the analysis, the validation performance is quite significant when compared to the existing methods, potentially ensuring the model isn’t overfitting, and making reliable predictions and decisions under uncertainty.

**Table 10:** Statistical analysis with 90% of training using CHB-MIT dataset.

Methods/ Metrics	Accuracy (%)			Sensitivity (%)			Specificity (%)			F1-Score (%)		
	Best	Mean	Variance	Best	Mean	Variance	Best	Mean	Variance	Best	Mean	Variance
RF^19^	83.77	69.45	112.54	79.72	62.01	172.70	70.36	65.05	38.72	74.75	63.13	84.60
GTDA-KNN^27^	92.44	85.74	23.73	93.20	84.71	37.53	95.14	87.99	113.19	94.16	86.12	62.01
PSO-ANN^20^	94.64	92.00	4.25	94.62	92.88	1.36	96.10	92.42	29.86	95.35	92.58	10.91
CL-ATBiLSTM^26^	94.64	92.00	4.25	94.62	92.88	1.36	96.10	92.42	29.86	95.35	92.58	10.91
DEF-DSVM ^28^	96.07	94.15	3.04	96.05	94.62	0.95	97.06	95.30	2.03	96.55	94.96	1.42
DCNN^17^	91.18	81.16	56.96	92.72	77.82	118.90	94.66	85.96	139.14	93.68	81.45	111.35
ForeSeiz^18^	93.69	90.32	5.80	93.67	91.61	1.87	95.62	90.02	94.46	94.63	90.56	34.25
SCFHSAN^21^	95.60	93.67	3.02	95.57	94.15	0.95	96.58	94.82	2.03	96.07	94.48	1.42
MLF-CNN^22^	96.55	94.62	3.05	96.52	95.10	0.95	97.54	95.78	2.03	97.03	95.43	1.42
SBTM	97.52	95.58	3.10	97.51	96.07	0.96	98.51	96.73	2.07	98.00	96.40	1.45

**Table 11:** Statistical analysis with 90% of training using Real-time dataset.

Methods/ Metrics	Accuracy (%)			Sensitivity (%)			Specificity (%)			F1-Score (%)		
	Best	Mean	Variance	Best	Mean	Variance	Best	Mean	Variance	Best	Mean	Variance
RF^19^	72.50	60.11	84.30	68.99	53.67	129.35	60.89	56.30	29.00	64.69	54.64	63.37
GTDA-KNN^27^	80.00	74.20	17.77	80.66	73.32	28.11	82.34	76.15	84.78	81.49	74.53	46.45
PSO-ANN^20^	81.91	79.62	3.18	81.89	80.38	1.02	83.17	79.98	22.37	82.52	80.13	8.17
CL-ATBiLSTM^26^	81.91	79.62	3.18	81.89	80.38	1.02	83.17	79.98	22.37	82.52	80.13	8.17
DEF-DSVM^28^	83.14	81.48	2.27	83.12	81.89	0.71	84.00	82.47	1.52	83.56	82.18	1.07
DCNN^17^	78.91	70.24	42.67	80.25	67.35	89.06	81.92	74.40	104.22	81.07	70.49	83.40
ForeSeiz^18^	81.09	78.16	4.35	81.07	79.29	1.40	82.75	77.91	70.75	81.90	78.38	25.66
SCFHSAN^21^	82.73	81.07	2.26	82.71	81.48	0.71	83.58	82.06	1.52	83.15	81.77	1.07
MLF-CNN^22^	83.56	81.89	2.29	83.53	82.30	0.71	84.41	82.89	1.52	83.97	82.59	1.07
SBTM	84.40	82.72	2.32	84.39	83.15	0.72	85.26	83.71	1.55	84.82	83.43	1.08

## Conclusion

In the proposed IoT-aided system for the prediction of epileptic seizures, the SBTM model is utilized for automated epileptic seizure prediction. Further, the utilization of the significant statistical, spectral-based, and Hjorth features retrieved using EEG data reduces the computational complexity of the proposed model. Further, the SBTM architecture excels in capturing the temporal patterns and nonlinear dynamics of EEG signals, making the model highly effective for predicting epileptic seizures. In addition, the Spizella Optimization is exploited for fine-tuning the hyperparameters of the classifier, resulting in enhanced prediction accuracy. More specifically, the incorporation of blockchain technology in the proposed approach prevents illegitimate access to sensitive medical data and assists in secure data sharing among the communication entities. Extensive experimentation is carried out, and the performance of the SBTM model is measured using specificity, sensitivity, and accuracy, and attained values of 98.51%, 97.51%, and 97.52% respectively. Further, the proposed system offers effective prediction and intimate the medical staff when the patient is experiencing a seizure, allowing timely intervention and treatment. Moreover, the proposed system effectively mitigates the challenges present in detecting real-time occurrences of seizures and serves as a valuable tool for epileptic seizure prediction. In the future, incorporating additional data sources and methodologies to improve prediction accuracy as well as data security. In addition, the research specifically addresses the critical metrics, including processing time, energy consumption on IoT devices, and end-to-end system performance.

## Data Availability

The dataset used to assess the effectiveness of the developed Spizella-based Bi-LSTM for disease prediction is done through the CHB-MIT dataset [16]. The dataset is collected from Children’s Hospital Boston, which consists of EEG recordings from young patients who had uncontrollable seizures.
